# The pattern of *Phosphate transporter 1* genes evolutionary divergence in *Glycine max* L.

**DOI:** 10.1186/1471-2229-13-48

**Published:** 2013-03-20

**Authors:** Chengming Fan, Xu Wang, Ruibo Hu, Yahui Wang, Chaowen Xiao, Ying Jiang, Xiaomei Zhang, Changying Zheng, Yong-Fu Fu

**Affiliations:** 1 MOA Key Lab of Soybean Biology (Beijing), National K’ey Facility of Crop Gene Resource and Genetic Improvement, Institute of Crop Sciences, Chinese Academy of Agricultural Sciences, 12 Zhongguancun Nandajie, Haidian District, Beijing, 100081, China; 2 CAS Key Laboratory of Biofuels, Shandong Provincial Key Laboratory of Energy Genetics, Qingdao Institute of BioEnergy and BioProcess Technology, Chinese Academy of Sciences, Qingdao, Shandong, 266101, China; 3 College of Agronomy and Plant Protection, Qingdao Agricultural University, Qingdao, 266109, China

**Keywords:** Phosphate transporter 1, Gene duplication, Gene divergence, Phosphorus homeostasis, Evolution, *Glycine max* L.

## Abstract

**Background:**

The *Phosphate transporter 1* (*PHT1*) gene family has crucial roles in phosphate uptake, translocation, remobilization, and optimization of metabolic processes using of Pi. Gene duplications expand the size of gene families, and subfunctionalization of paralog gene pairs is a predominant tendency after gene duplications. To date, experimental evidence for the evolutionary relationships among different paralog gene pairs of a given gene family in soybean is limited.

**Results:**

All potential *Phosphate transporter 1* genes in *Glycine max* L. (*GmPHT1*) were systematically analyzed using both bioinformatics and experimentation. The soybean *PHT1* genes originated from four distinct ancestors prior to the Gamma WGT and formed 7 paralog gene pairs and a singleton gene. Six of the paralog gene pairs underwent subfunctionalization, and while *GmPHT1;4* paralog gene experienced pseudogenization. Examination of long-term evolutionary changes, six *GmPHT1* paralog gene pairs diverged at multiple levels, in aspects of spatio-temporal expression patterns and/or quanta, phosphates affinity properties, subcellular localization, and responses to phosphorus stress.

**Conclusions:**

These characterized divergences occurred in tissue- and/or development-specific modes, or conditional modes. Moreover, they have synergistically shaped the evolutionary rate of *GmPHT1* family, as well as maintained phosphorus homeostasis at cells and in the whole plant.

## Background

To adapt to challenging environments, plants have developed dramatic modifications in morphological, physiological, biochemical and molecular processes. Gene duplications are widespread in plant genomes, having accumulated a wealth of genetic raw materials to meet the selection pressures of new environmental conditions [1-5]. After gene duplications, there are several possible fates for duplicated genes (or paralogs), which include subfunctionalization through purifying selections (Ka/Ks < 1) [6], neofunctionalization through positive selections (Ka/Ks > 1) [7], pseudogenization [8], and loss in genome (fractionation) [9-11]. Subfunctionalization is the predominant paralog outcome following duplications [12]. It reduces the fitness cost of gene duplication by buffering dosage imbalances, as well as maintaining the functional requirements of the ancestral locus [13].

In plants, phosphorus is one of three primary mineral nutrients. It is second most limiting macronutrient for optimal growth, due to the relatively large amounts of Pi required by plants, the limited amount of available phosphorus (orthophosphate, Pi), and the poor mobility of phosphorus in soil [14,15]. Plant uptake of Pi from soil relies heavily upon the phosphate transporter 1 family (PHT1) [16]. *PHT1* genes code for plasma membrane proteins, which contain 12 transmembrane domains. The PHT1 proteins are functionally involved in Pi uptake from the soil, Pi translocation across plant tissues, and Pi remobilization from senescent organs (Review in [14,17,18]). Homologous genes of *PHT1* have been identified in a wide range of species, and they share conserved functions in the Pi uptake (Additional file 1) [14] and variable Pi affinity [19-22].

The soybean has experienced the Gamma whole genome triplication (Gamma WGT) ~130 to 240 million years ago (mya), the legume WGD (Legume WGD, ~58 mya), and the *Glycine* WGD in the *Glycine* lineage (*Glycine* WGD, ~13 mya) [23,24]. As a result, in the present soybean genome, about 75% of the genes have multiple paralogs [23,25]. Approximately 50% of these paralogs were differentially expressed and underwent subfunctionalization [12], possibly contributing to phenotypic variation in polyploids [3].

Many *PHT1* genes have functionally identified in many plants, but they are studied as an individual. This was a limiting step for both further functional characterization of PHT1 family as a whole and genetic evolution analysis of them in relation to low Pi environment adaptations. In this study, 15 *GmPHT1* (***G****lycine****m****ax****PHT1***) family paralogs from soybean were identified. Based on data from spatio-temporal expression profiles, functional characterizations in a heterologous yeast system and subcellular localizations, we propose fates of paralogs of soybean *PHT1* were subfunctionalization. These results provided a strong basis for function analysis and evolution of gene families.

## Results

### Identification, phylogenetic relationship and promoters of soybean *PHT1* genes

Fourteen soybean *PHT1* genes with full length sequence were found [26,27]. In addition, a syntenic analysis using the PGDD or CoGe databases identified one potential pseudogene (Glyma13g18420), which had a truncated open reading frame length (ORF) of 444 bp. This potential pseudogene, which was determined to be masked, has not been previously identified [21,26,28]. For an unified nomenclature for the soybean *PHT1*s (Additional file 2), fifteen soybean *PHT1* genes were renamed as *GLYma;Pht1;1* through *GLYma;Pht1;15* according to the Commission for Plant Gene Nomenclature, and abbreviated as *GmPHT1;1* through *15* in the following content.

**Figure 1:**
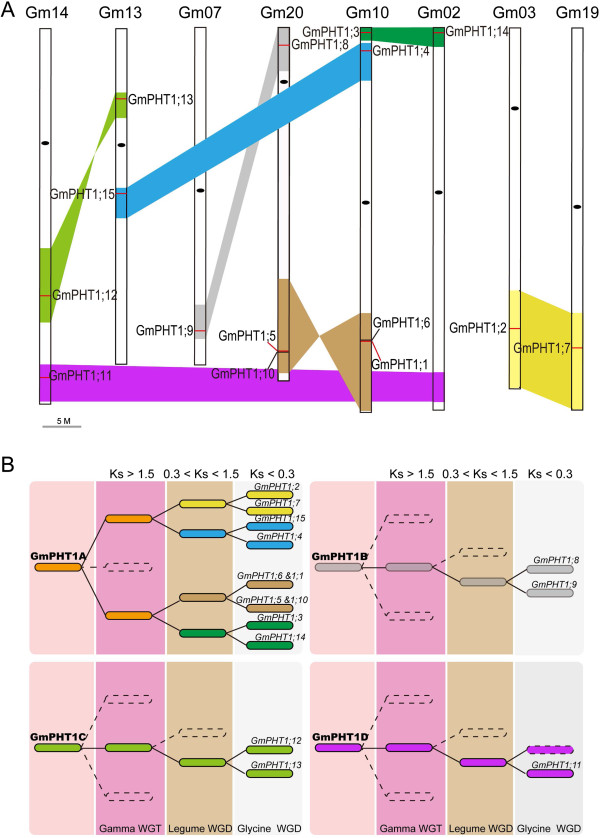
**The evolution of the *GmPHT1 *gene family. A**, Syntenic relationships among homologous blocks carrying the 15 *GmPHT1* sequences after the *Glycine* WGD event. Similar colored blocks imply homology, the short red lines within these blocks show the location of *GmPHT1*s, and the black oval is the centromere. *GmPHT1;15* is the pseudogene and the paralog gene of *GmPHT1;11* was lost. **B**, The evolutionary model for *GmPHT1-*containing genomic blocks in the process of the soybean genome evolution, indicating *GmPHT1*s originated from four independent ancestors. Different backgrounds depict different whole genome duplication events. The colored blocks imply homology based on the average *Ks* values. The paralog gene of *GmPHT1;11* was lost in the dotted block dotted, although other genes are collinear with the block containing *GmPHT1;11*. The detailed collinearity relationships are shown in Additional file 5C.

These 15 *PHT1* genes are dispersed across eight chromosomes and form 7 paralog gene pairs and one singleton (Figure 1A). These paralog gene pairs shared 93.5 ~ 97.1% in sequence (Additional file 3A) and similar gene structures (Additional file 3B). For example, both *GmPHT1;8* and *GmPHT1;9* have 3 exons, both *GmPHT1;3* and *GmPHT1;14* are composed of 2 exons, while the others only contain one. However, an extra intron in 5^′^-UTRs of *GmPHT1;1*, *GmPHT1;4* and *GmPHT1;5* was identified (Additional file 3B).

To identify the phylogenetic relationship of the soybean *PHT1* genes with full length sequence, a neighbor-joining tree was reconstructed based on the multiple sequence alignment (Additional file 4). The PHT1 family was monophyletic [29] and can be grouped into four subfamilies in the angiosperm: the subfamily I is composed of *PHT1* genes induced by arbuscular mycorrhizal fungi (AMF), subfamily II genes are from both mono- and dicotyledonous species, subfamily III are exclusively from dicotyledonous species, and subfamily IV are exclusively from monocotyledonous species [26,30]. Furthermore, the subfamily II was closely related to the homologs from the fungi by high bootstrap value (Additional file 4), indicating it was present before the occurrence of terrestrial plant and an older evolutionary lineage. In the soybean, fourteen PHT1 genes were clustered into three subfamilies: subfamily I, subfamily II and subfamily III (Additional file 4) [21,26].

The paralog gene pair’s promoter region sequence similarities are lower than their CDS sequences (Additional file 3A). Many similar *cis*-acting regulatory DNA elements, which are relative to the nodulin, root, flower, leaf, seed, abiotic or biotic stress, sugar and hormone (Additional file 5A), can be found in promoter regions of 14 *PHT1* genes according to the PLACE results [31]. For example, the *cis*-elements relative to the root-specific (ROOTMOTIFTAPOX1) and nodule-specific (NODCON1GM and NODCON2GM) are present in 14 soybean *PHT1* promoters (Additional file 5A). Except *GmPHT1*;*9* promoter, other soybean *PHT1* promoters contain phosphate starvation responsive *cis*-element (PIBS). And *GmPHT1*;*9* promoter did not embody PIBS motif but a variant PIBS motif (76% similarity to PIBS) (Additional file 5A). The differences in common *cis*-elements across these promoter regions include both their number and their distance from the starting code (Additional file 5A). That indicated the number of *cis*-elements and their distance from the transcription start sites affected response abilities of PHT1 to the environment.

### Soybean PHT1 genes originated from four ancestors prior to the Gamma WGT event

The average synonymous substitution rate (*Ks*) of homologous blocks is a function of genomic evolutionary events that occurred since two homologous blocks diverged from a common ancestor [25,32]. The modern soybean genome has undergone Gamma WGT (*Ks* > 1.5), Legume WGD ( *Ks* ~ 0.3 to 1.5) and *Glycine* WGD (*Ks* ~ 0 to 0.3) [24,25]. To analyze the soybean *PHT* gene duplication relationship, *Medicago truncatula*, which experienced the Gamma WGT event and Legume WGD event [33], was selected as the reference gene order and 9 *Medicago PHT1*s were identified (Additional file 5B).

Based on the analysis of homologous genomic regions, seven paralog *GmPHT1* gene pairs fell into 14 syntenic blocks and diverged after the Glycine WGD event. This was based on each pair of blocks having collinearity, with the average *Ks* ranging from 0.19 to 0.25 (Figure 1A, Additional file 5C). Although the paralog gene of *GmPHT1;11* was lost in the syntenic block of chromosome 2, the block containing *GmPHT1;11* underwent *Glycine* WGD events (Figure 1A and Additional file 5C). Because the homologous block containing *MtPHT4* on Chromosome 5 in *M. truncatula* had a collinearity with the block harboring *GmPHT1;11* (Additional file 5B). With the exception of the WGD duplication, the ancestor of two paralog gene pairs, *GmPHT1;1*/*5* and *GmPHT1;6*/*10,* experienced tandem duplication before the Legume WGD event. Because the blocks embodying *GmPHT1;1*/*6* and *GmPHT1;5*/*10* had a collinearity with the block containing two tandem genes, *MtPHT5* and *7,* on Chromosome 1 of *M. truncatula* (Additional file 5B).

Based on the average *Ks* values for the homologous blocks (Additional file 5C), the evolution history of all 15 *PHT1* genes was predicted. These *PHT1* genes were categorized into four subgroups, GmPHT1A through D (Figure 1B). For example, GmPHT1A included eight syntenic blocks, which represented five paralog gene pairs of *PHT1* genes, *GmPHT1;2*/*7*, *GmPHT1;4*/*15*, *GmPHT1;1/5*, *GmPHT1;6*/*10* and *GmPHT1;3*/*14*. Both GmPHT1B and GmPHT1C contained two syntenic blocks and each contained one paralog gene pair of *PHT1* genes, GmPHT*1;8*/*9* and *GmPHT1;12*/*13*, respectively. GmPHT1D group was made up of two syntenic blocks and contained the *GmPHT1;11* gene. These results indicated the soybean PHT1 gene family originates from four distinct ancestors, at least prior to the Gamma WGT event.

### Divergence of *GmPHT1* expressions in different tissues and in developmental stages

**Figure 2:**
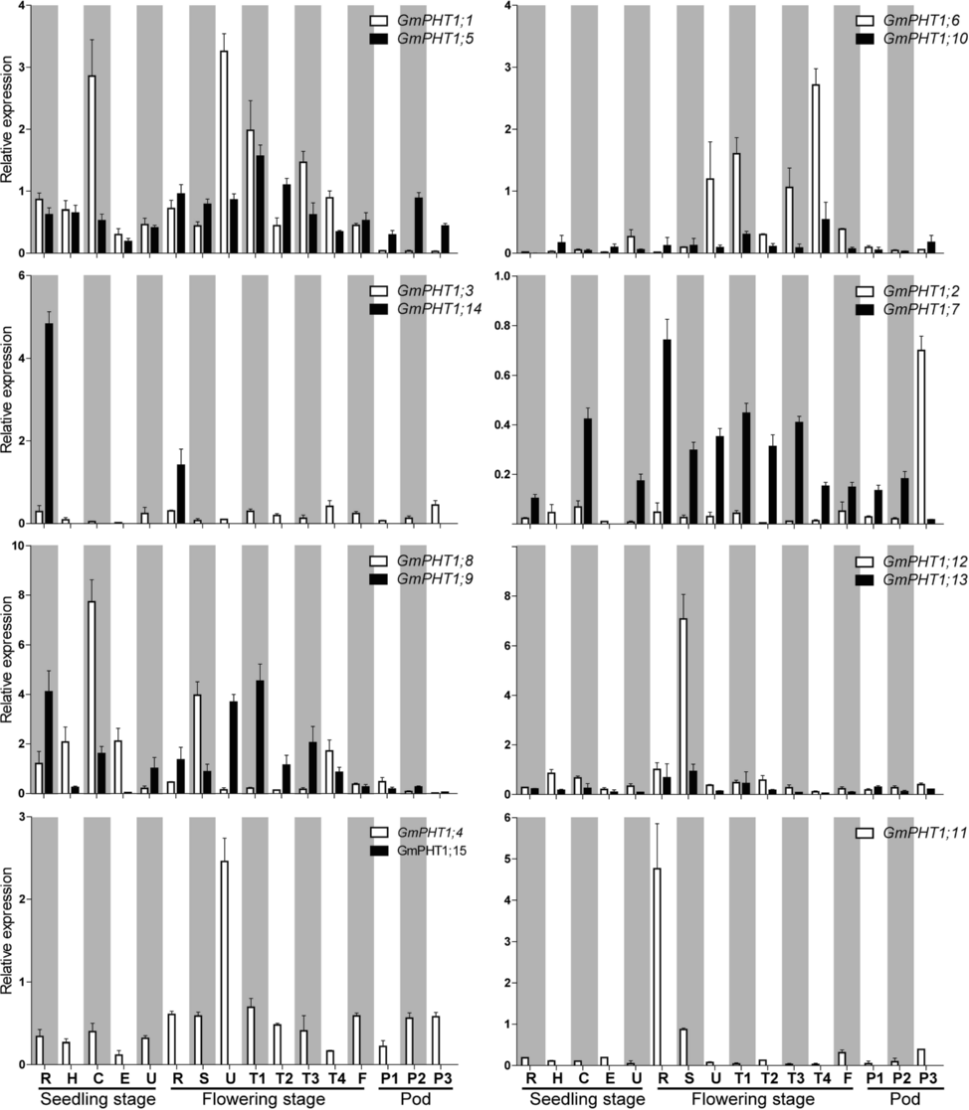
**The spatio-temporal transcription of *GmPHT1.*** R, H, C, E, U, S, T1, T2, T3, T4, F: the root, hypocotyl, cotyledon, epicotyls, unifoliolate leaf, the stem, the first trifoliolate leaf, the second trifoliolate leaf, the third trifoliolate leaf, the fourth trifoliolate leaf and the flower, respectively. And P1, P2, and P3: seven, fourteen and twenty one days after the onset of flowering, respectively. The geometric means of *GmSKIP16* and *GmUNKI* transcripts were used as reference transcripts. The values are means of three replicates and each replicate represented a pool from at least five plants. Error bars represent SD.

Duplicated genes typically exhibit increased expression divergence, thus gene expression changes shape evolutionary rates of proteins and re-establish the gene balance after duplication [34]. Based on the spatio-temporal expression of *GmPHT1* genes through real time quantitative RT-PCR (qRT-PCR), most paralog gene pairs co-expressed in 16 tissues (Figure 2, Additional file 5D). However, two paralog gene pairs demonstrated obvious expression pattern differences. *GmPHT1;14,* was only expressed roots, but the expression of its paralog gene, *GmPHT1;3*, was detected at low level in all samples. And the *GmPHT1;2* paralog gene, *GmPHT1;7*, did not express in hypocotyls and epicotyls at the seedling stage.

**Figure 3:**
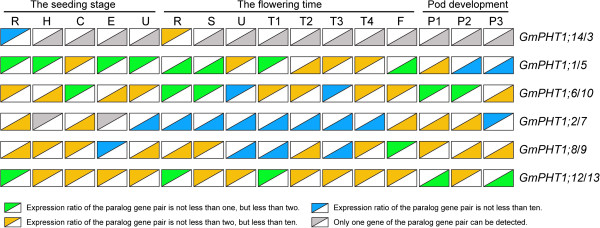
**The transcript divergence of six paralogous pairs of genes within a given tissue in different developmental stages.** The upper triangles showed the expression of the lift genes of the paralog gene pairs, and the lower triangle the expression of the right genes of the paralog gene pairs. The raw average relative expressions were in the Additional file 5D. R, H, C, E, U, S, T1, T2, T3, T4, F: the root, hypocotyl, cotyledon, epicotyls, unifoliolate leaf, the stem, the first trifoliolate leaf, the second trifoliolate leaf, the third trifoliolate leaf, the fourth trifoliolate leaf and the flower, respectively. And P1, P2, and P3: seven, fourteen and twenty one days after the onset of flowering respectively. The geometric means of *GmSKIP16* and *GmUNKI* transcripts were used as the reference transcript.

The expression levels among paralog gene pairs demonstrated clear divergence (Figure 3). Among the six paralog gene pairs, 80 pairs of co-expression values were obtained from 16 tissues. The overall expression levels difference were as follows, ~24% had between a 1 to 2 fold change, ~ 54% between a 2 -to 10 fold change, and ~ 22% had more than a 10-fold change. Moreover, gene-biased expression levels between a paralog gene pair were observed. For example, one paralog gene pair, *GmPHT1;2* and *GmPHT1;7* displayed expression biased to *GmPHT1;2* in 15 tissues, only different in the pods after 21 FAD. In addition, expressions were biased to GmPHT1;12 between the paralog gene pair, *GmPHT1;12* and *GmPHT1;13* (Figure 3).

### *GmPHT1* genes differential response to the Pi stress

**Figure 4:**
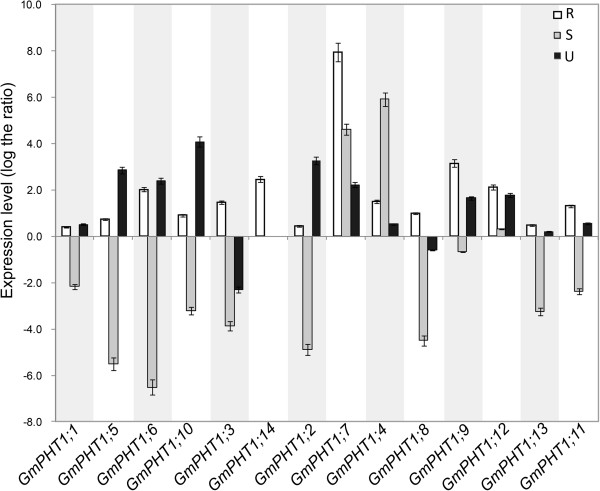
***GmPHT1 *transcription in the response to the low Pi (1 μM) stress.** At least five individual plants per treatment were harvested when the unifoliolate leaves fully expanded. The transcript abundance of *GmPHT1* genes in the roots (R), stems (S) and unifoliolate leaves (U) were shown using the expression of the 500 μM Pi treatment group as a control. The geometric means of *GmSKIP16* and *GmUNKI* transcripts were used as the reference transcript. The values are means of three replicates and each replicate represented a pool from at least five plants. Error bars represent SD.

Plant root performance dependents directly on Pi availability in soil [35]. Pi stress induces most of the known *PHT1s* genes (Review in [14,17,18]). To investigate *GmPHT1* responses under low Pi (Pi = 1 μM) stress conditions, soybean PHT1 gene expressions were evaluated in the root, stem and leaf, at the vegetative stage (Figure 4). Compared with expressions under high Pi (Pi = 500 μM) conditions, all soybean *PHT1* genes were up-regulated in roots under the low Pi condition. Divergent expressions of some paralogs were observed in stem or leaf tissues (Figure 4). Except *GmPHT1;4*, *7* and *12*, transcriptions of other soyben *PHT1* genes were down-regulated in stems under low Pi conditions. Additionally, expressions of *GmPHT1;3* and *8* were induced by the high Pi condition in leaves.

**Figure 5:**
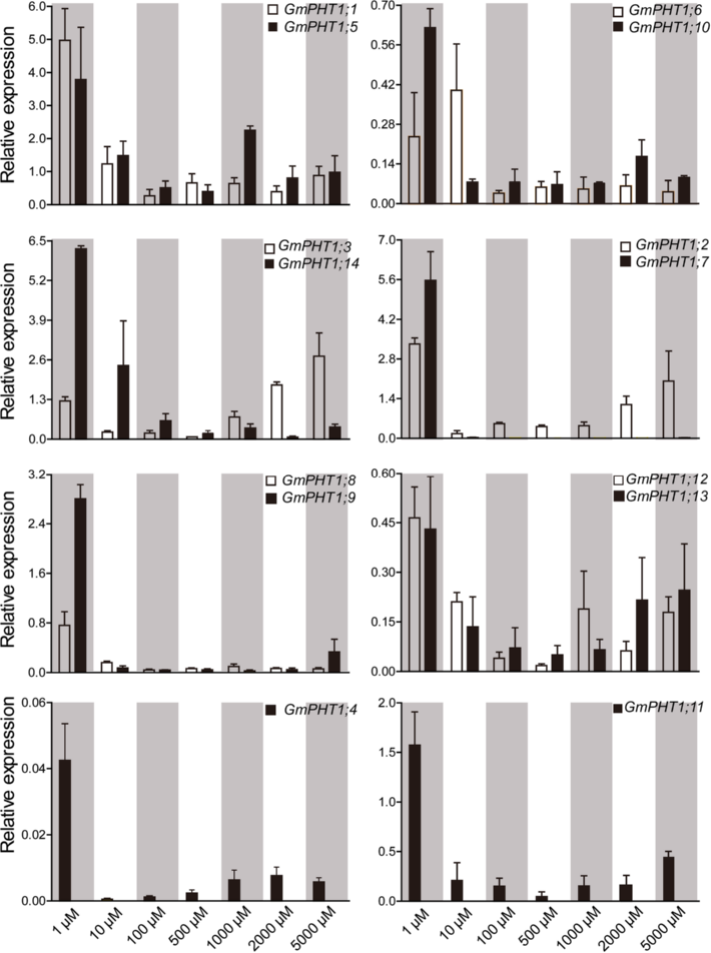
***GmPHT1 *transcription in the root is affected by external Pi concentration.** Roots of at least five individual plants per treatment were harvested when the unifoliolate leaves fully expanded. Different paralogous pair genes were plotted in individual figures. The geometric means of *GmSKIP16* and *GmUNKI* transcripts were used as the reference transcript. The values are means of three replicates and each replicate represented a pool from at least five plants. Error bars represent SD.

In addition to under the low Pi condition, the responses of *GmPHT1* genes to a series of Pi concentrations in the roots were employed to investigate the expression divergence of the paralog gene pairs (Figure 5). Compared with those under the low Pi (Pi = 1 μM) condition, expressions of all 13 *GmPHT1* genes were significantly supressed in the roots under the Pi = 10 μM condition except *GmPHT1;6*. And when the external Pi concentration was more than 10 μM, the range of expressions were very narrow except *GmPHT1;2*, *GmPHT1;3, GmPHT1;12* and *GmPHT1;13*. That indicated most paralog gene pairs showed the similar responses to the external Pi in the root except *GmPHT1;2*/*7* and *GmPHT1;3*/*14*. And under the high-Pi condition, the transcriptions of *GmPHT1;2* and *GmPHT1;3* were induced, but the strength were lower than under the low Pi condition (Figure 5). That suggested that *GmPHT1;2* and *GmPHT1;3* may contributed to Pi tolerance in soybean.

### Divergence in the Pi transport activities of GmPHT1 in yeast

To investigate the divergence of 14 soybean *PHT1s* in the Pi transport ability, the abilities of heterologous complementation of yeast double mutant (PAM2, *Δpho84Δpho89*) [36] were tested on the nutrition defect media (Additional file 6). The resulting sequences were confirmed by sequencing and cloned into pYES-DEST52 drive by *GAL1* promoter.

**Figure 6:**
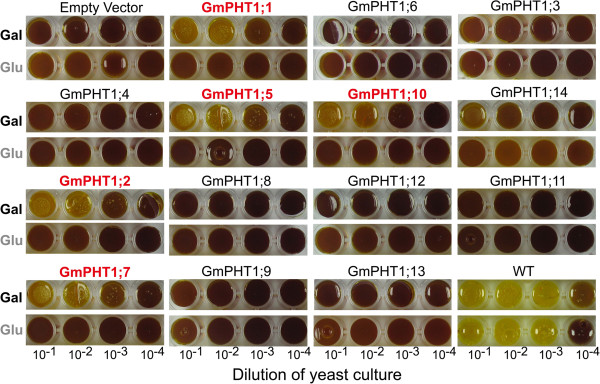
**Complementation analysis of *GmPHT1 *genes in the yeast double mutant PAM2.** Wild type (WT) and mutant (PAM2) yeast strains containing either an empty pYES-DEST52 vector (Empty vector) or pYES-DEST52 (bearing one of the *GmPHT1* sequences). Serial dilutions were spotted onto a selective medium supplemented with low concentrations (10 μM) of Pi, with either glucose (Glu, non-induced medium) or galactose (Gal, induced medium) as the carbon source. Each spot represented 5 μl yeast culture, diluted from a master culture, as indicated. Yellow color indicates the cell in the spots grew well. Images were captured after three days.

As Figure 6 shown, only one paralog gene pairs, *GmPHT1;6/10*, showed difference of complementation ability. And PAM2 cells carrying *GmPHT1;1*, *GmPHT1;2*, *GmPHT1;5*, *GmPHT1;7*, and *GmPHT1;10* grew well on the induced modified SD media (the carbon source is galactose) under the low Pi condition, whereas PAM2 cells harboring other 9 *GmPHT1* and the empty vector did not grow normally under the same conditions. This data suggested that *GmPHT1;1*, *GmPHT1;2*, *GmPHT1;5*, *GmPHT1;7*, and *GmPHT1;10* may be high-affinity phosphate transporters and others were lower-affinity ones.

**Figure 7:**
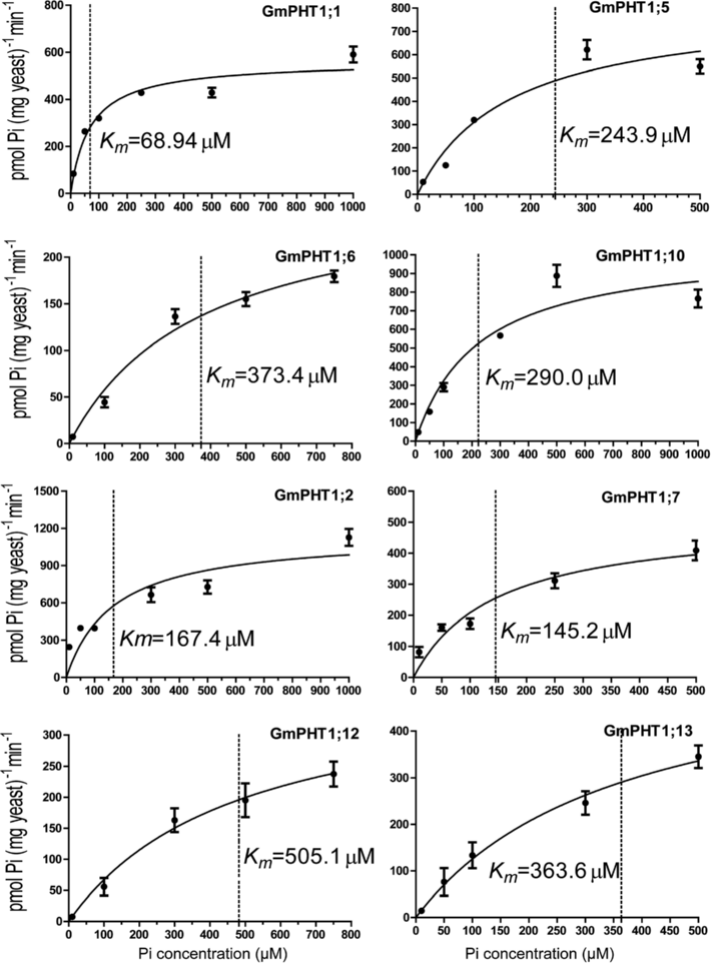
**Radioactive phosphorus (**^**32**^**Pi) uptake by PAM2 cells carrying a*GmPHT1 *gene.** Each point represents the average and SD of at least three uptake experiments. The concentration of external Pi was 10 μM, 50 μM, 100 μM, 300 μM, 500 μM or 1000 μM. *Km* values are indicated next to the dot lines and gained through GraphPad Prism 5.

Kinetic parameters (*Km*) can display the affinity ability of the PHT1 proteins for transporting Pi. Subsequent ^32^Pi uptake assays were employed to further confirm the different affinity of GmPHT1 and to analyze *K*_*m*_ values of Pi uptake of 4 paralogous pair transporters (Figure 7). The paralogous gene pairs also displayed divergence on the affinity for Pi. For example, GmPHT1;1 had a *K*_*m*_ of 68.9 μM, while its papralogous transporter GmPHT1;5 had a *K*_*m*_ of 243.9 μM; the *K*_*m*_ of GmPHT1;12 was 505.1 μM, whereas the *K*_*m*_ of GmPHT1;13 was 363.6 μM, both of them were low affinity transporters.

### Divergence on the subcellular localization of GmPHT1 proteins

**Figure 8:**
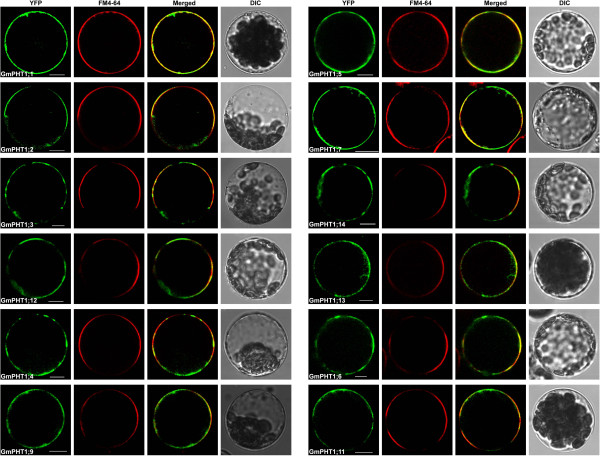
**Transient transcription in *A. thaliana *mesophyll protoplasts of 12 *GmPHT1-YFP *fusions.** The FM4-64 signal is diagnostic for the plasma membrane. DIC: differential interference contrast. Scale bar: 10 μm.

The PHT1 protein in plants primarily localizes to the plasma membrane [14,20,37]. Under Pi stress they are targeted to endocytic compartments [38]. To analyze subcellular localization of GmPHT1 proteins, we tagged the GmPHT1 proteins with yellow fluorescence protein (YFP) at their C-terminal. With the exception of GmPHT1;8 and GmPHT1;10, twelve GmPHT1 proteins co-localized to the plasma membrane (Figure 8).

**Figure 9:**
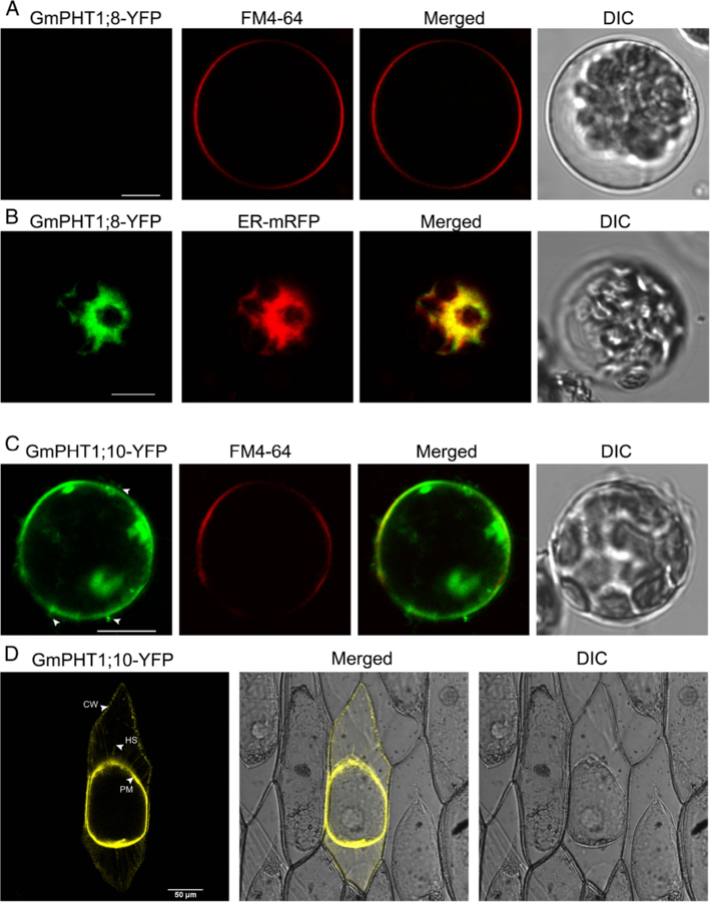
**Sub-cellular localization of GmPHT1;8 and GmPHT1;10 in *A. thaliana *protoplasts. A**, No signal of GmPHT1;8-YFP on plasma membrane. FM4-64: plasma membrane marker. **B**, The co-localization of GmPHT1;8-YFP with the ER specific marker mRFP. **C**, The subcellular localization of GmPHT1;10-YFPAnd FM4-64: plasma membrane marker. Arrow heads display extracellular signals. **D**, The localization of GmPHT1;10 in plasmolyzed onion epidermal cells. CW: cell wall, HS: Hechtian strands, PM: plasma membrane. Scale bar: 10 μm for **A**, **B**, and **C**, 50 μm for **D**.

One exceptional case was the localization pattern of GmPHT1;8. Though no fluorescent signal of GmPHT1;8-YFP was detected in the plasma membrane (Figure 9A), a strong signals in endoplasmic reticulum (ER) was detected. GmPHT1;8 co-localized to the ER in *Arabidopsis* mesophyll protoplasts along with ER-marker [39] (Figure 9B).

Another exception was GmPHT1;10 localization. Here, GmPHT1;10 fluorescent signals were detected both at the plasma membrane as well as outside of the plasma membrane (Figure 9C). These localization patterns were recapitulated in plasmolytic onion epidermal cells. Strong fluorescence was detected in cell walls, plasma membranes and Hechtian strands (Figure 9D). Moreover, paralog gene, *GmPHT1;6*, and another gene pair, *GmPHT1;1* and *5*, presented with strong signals only in the plasma membrane (Additional file 7). This pattern was a copy of that found in *Medicago* MtPT3 [20] and *Arabidopsis* Pht1;1 [37].

## Discussion

### Different lineages of PHT1 genes undergoing different nature selections during the plant evolutionary process

Land plants only gain phosphorous from soil solutions through the root, and most *PHT1* genes express and are induced by the Pi starvation or by AMF in the root [16,40,41], indicating Pi uptake is heavily dependent on the phosphate transporter 1 family in the plant. In order to adapt the low-Pi environment and improve Pi uptake, two Pi uptake pathways have evolved under the nature selection. First, the direct Pi acquisition pathway acts through modifications of root architecture, root length and lateral root numbers [42-44]. The second pathway is symbiotic Pi uptake, which acts through plant/fungi interactions [29,45].

Before the occurrence of the first terrestrial plant, which were present about 475 million years ago [46], the PHT1 subfamily II has been divergent based on the phylogenic tree (Additional file 4), indicating the direct Pi acquisition pathway was the main Pi acquisition pathway of ancient plants. About 460 million years ago, AMF occurred and may have played a crucial role in facilitating the colonization of land by plants most likely only consisted of plants on the bryophytic level [47]. And then the PHT1 subfamily I was diverged, suggesting the subfamily I is another older evolutionary lineage. Therefore, AMF have been symbionts of land plants for at least 450 million years old, and the symbiotic Pi uptake is an evolutionarily ancient Pi acquisition strategy for plant life on land [29,48].

In the evolutionary process of plants, genome duplications were ancient and recurrent [49]. They provide the important raw genetic material to adapt to challenging environment and increase the diversity of plants. New genome sequences and improved analytical approaches are clarifying angiosperm evolution and revealing patterns of differential gene loss after genome duplication and differential gene retention associated with evolution of some morphological complexity [50]. According to the evolution of the PHT1 family, the members of PHT1 subfamily I and II, which are diverged eailier, were not expanded as expected in angiosperms compared with the subfamily III and IV. For example, In Arabidopsis, which is not host plant of AMF and experienced at least three polyploidy events [51], nine members of the PHT1 family were found, but no members of PHT1 subfamily I [52,53]. In *Populus trichocarpa*, experiencing at least two polyploidy events [54], *PtPT10* and *PtPT8* belong to the PHT1 subfamily I, and *PtPT8* is a pseudogene [30]. In the rice, experiencing one polyploidy event [55], contain two nonredundant members of the PHT1 subfamily I, *OsPT11* and *OsPT13*[56,57]. And *OsPT13* is conserved and special across monocotyledons [56]. In the soybean, undergoing three WGD events [23-25], three members of the PHT1 subfamily I, *GmPHT1;11*, *12* and *13*, were found. *GmPHT1;12* and *GmPHT1;13*, were a paralog gene pair arisen after the *Glycine* WGD event. And the *GmPHT1;11* paralog gene was lost after the Legume WGD event and then *GmPHT1;11* became a singleton (Figure 1). Conversely, the GmPHT1-A experienced three rounds of WGD events and is composed of 9 members with whole coding sequences, plus one pseudogene (*GmPHT1;15*). For the PHT1 subfamily II, about two members can be found in each plants, while members of subfamily III or IV were expanded after gene duplication (Additional file 4). Taken together, different selection pressures retained different subgroups during the plant evolution.

### Multiple divergences resulted in retention of paralogs from one gene family

Polyploidy is widespread and is a process that recurrently shaped eukaryotic genomes in plant. After genome duplication once fixed within species, the three possible fates of duplicated genes: neofunctionalization, subfunctionalization or nonfunctionalization [6-8,58]. If the Ka/Ks value is more than 1.0, gene copies would undergo positive selection and have new functions. On the contrary, subfunctionalization would be expected to undergo purifying selection. The Ka/Ks values for all soybean PHT1 genes were less than 1.0 (Additional file 5F), thus, the paralog *PHT1* gene pairs were undergoing purifying selections in the soybean evolution and subfunctionalized. In the soybean genome, about 75% genes are present in multiple copies, and approximately 50% of paralogs are differentially expressed and have undergone expression subfunctionalization, and only a small proportion of the duplicated genes have been neofunctionalized or non-functionalized [12], suggesting that the main fate of duplicated genes were subfunctionalization.

Although the different functions of Pi transport were that they have different affinities, all the published *PHT1* genes display conserved functions of Pi transport. In one species, the greatest difference is the divergence of their expression profile, which results in their differently functional sites in the plant. In Arabidospsis, *AtPHT1;6* expresses only in flowers, and both *AtPHT1;8* and *9* express only in the roots [52], and transcriptions of *AtPHT1;5* are detected in the old tissues and induced by ethylene [59]. Additionally, most *AtPHT1* genes exhibit strong expression in several tissues although their expressions overlapped to some extent [16]. In angiosperms, some members of the PHT1 family, such as *MtPT4, OsPT11*, *OsPT13*, *TaPHT1*, *HvPT8*, *StPT4*, *LePT4* and *PtPT10*, are induced only by AMF, while transcriptions of other members, such as *StPT3*, *OsPT1*, *OsPT2*, *OsPT3*, *OsPT6*, *OsPT9*, and *OsPT10* are not special to AMF [30,57,60-64]. The response of most PHT1 genes to the low Pi are similar, but to deficiencies of other nutrient elements, such as nitrogen, potassium, iron, and zinc, are different [27,65]. Three different paralog gene pairs, show different expression patterns or levels under deficient N, K, or Fe conditions [27], suggesting another subfunctionalization event among these paralogs.

Subfunctionalization can be taken as genetic redundancy [66,67]. For example, in *Arabidopsis*, the Pi uptake rates of single mutants, *pht1;1* and *pht1;4,* were reduced about 20%, but the rate of double mutant was reduced approximately 57%, under the low-Pi condition [37]. Mutations in tomato (*Solanum lycopersicum*) *StPT4* affected neither mycorrhizal Pi uptake nor establishment of the symbiotic pathway, this was due to arbuscular-mycorrhizal symbiosis triggering expression of other three *PHT1* genes [64]. These results indicate Pi uptake functionally redundancy. The expression profiles of soybean *PHT1* (Figure 2), indicates extensively overlaid, these results suggest redundancy in these paralogs as well.

PHT1, as a plasma membrane protein, consists of two regions of six transmembrane domains separated by a hydrophilic loop [68,69]. All the 14 soybean PHT1 proteins, like other PHT1s, contain 12 transmembrane domains (Additional file 8). Up to now, the PHT1 family members were believed to localize finally to the plasma membrane (the function site) after post-translational modifications [38,70]. According to our results (Figure 8), the fluorescence signals of most soybean PHT1s were detected in the plasma membrane. Except the plasma membrane, the fluorescence signals of MtPT3 [20] and AtPHT1;1 [37] can be detected in hechtian strands. The subcellular localization of GmPHT1;10 in onion epidermal cells was similar to that of MtPT3 and AtPHT1;1 (Figure 9D), but the same localization pattern was not observed in its paralog (Additional file 9). Interestingly, the localization of GmPHT1;8 was not at the cytoplasmic membrane but at the endoplasmic reticulum (Figure 9B), however its paralog, GmPHT1;9, localized to the plasma membrane.

### Subfunctionalization of paralogs beneficial to soybean Pi uptake, translocation and remobilization

To enhance acquisition of external Pi, plants can morphologically regulate root architecture to enhance the root surface/soil volume ratio, also root architecture is closely related to P efficiency [40,71-74]. The number and length of lateral roots and the length of primary roots increased under the low-Pi condition (Additional file 9). At the molecular level, the members of PHT1 family play important roles in Pi uptake from soil solutions thus exhibiting robust expression in roots [16,40]. According to our results (Figure 4) and others’ [27], fourteen soybean *PHT1* genes expressed in the root were up-regulated by low-Pi stress. Moreover, GmPHT1;1, 2, 5, 7, and 10 had high affinity to Pi. Thus, GmPHT1;1, 2, 5, 7, and 10 may play important roles in the direct Pi uptake from low-Pi soil solutions.

In addition to direct Pi uptake, symbiotic phosphate uptake is an ancestral Pi acquisition strategy for plants, meaning that some *PHT1* genes are induced by arbuscular mycorrhizas [16,29,40,63,75]. In the soybean, the transcription of three soybean *PHT1* genes, *GmPT7*, *10* and *11* (*GmPHT1;11*, *13*, and *12*, respectively in this study), was induced by arbuscular mycorrhizal fungi [26]. These results indicate these three genes have important roles in soybean symbiotic Pi uptake.

After Pi uptake, distribution and remobilization of Pi, within the plant, is accomplished through the membrane transport systems of the shoots to the sink tissues wherever symplastic connections are lost [15,40]. Although other PHT gene families, such as the PHT2 family [76], are involved the process, the PHT1 gene family localization in the plasma membrane is the most important [19,52,77,78]. Based on our results, *GmPHT1;7*, *8*, *10* and *12* exhibited stronger expression than their corresponding paralog genes in the stems, at the seedling and flowering stage (Figure 2), suggesting involvement in internal Pi transport in the shoots.

Another developmental signal, senescence, has been reported to strongly induced expression of some *PHT1* genes [30,59,79], and most leaf phosphorus is remobilized to the seed during reproductive development in soybean [80]. For instance, in other plants, the expression of *PhPT1* is up regulated during petunia petal senescence [79] and transcript level of *Pht1;5* is elevated in the old leaves in *Arabidopsis*[59]. In the soybean, *GmPHT1;1* expression increased in unifoliolates along with developmental process and reached the peak during flowering time (Figure 2). Moreover, again during flowering time, the transcription level of *GmPHT1;1* was relative to leaf ages (Figure 2).This suggests that *GmPHT1;1* is related to the re-utilization of Pi from older leaves. Cotyledons are the main phosphorus store tissue and the phosphorus resource tissue at the seedling stage. High expression of *GmPHT1;8* was detected in the cotyledons at the seedling stage (Figure 2), suggesting *GmPHT1*;*8* played an important roles in recycling Pi from cotyledons.

Different subcellular localizations of GmPHT1 proteins correlated to cellular homeostasis according to our findings. Although the majority of GmPHT1 proteins localized on cytoplasma membrane (Figure 8), similarly to PHT1s in other plants [81], GmPHT1;8 and GmPHT1;10 have unique localization patterns. GmPHT1;10 localized to Hechtian strands in addition to the cytoplasma membrane and cell walls. These Hechtian transporters may play critical roles in Pi transport between the cytoplasmic membrane and cell wall or between cells [82]. Perhaps, GmPHT1;10 had important roles in the cross talk of Pi flux or signals amongst the cells. In addition, GmPHT1;8 localized, exclusively, to the endomembrane system instead of cytoplasma membrane (Figure 9B). A functional auxin transporter, AtPIN5, does not have a direct role in cell-to-cell transport but regulates intracellular auxin homeostasis and localizes to endoplasmic reticulum (ER), unlike other characterized plasma membrane PIN proteins [83]. Given the function of the AtPIN5, this result may indicate GmPHT1;8 has a role in regulating intracellular Pi homeostasis and metabolism.

## Conclusion

In the soybean, there were 14 *PHT1* genes with full whole CDS plus one pseudogene, and they originated from four different different ancestors, GmPHT1A, B, C and D, before the Gamma WGT events in the soybean evolution history. Three polyploidy events expanded the members of GmPHT1A. In addition, one tandem duplication also increased the members of GmPHT1A after the Legume WGD and before the *Glycine* WGD. The retentions of paralog genes of GmPHT1B and C were only after the *Glycine* WGD. GmPHT1D contained one member, of which paralog gene was lost. Fourteen soybean *PHT1*s underwent the purifying selection and had the conserved function in Pi uptake although they had different affinities for Pi, and *GmPHT1;15* experienced pseudogenization. Expression divergence levels were the main style of subfunctionalization of the paralog gene pairs. The expression ratios were more than two amongst paralog gene pairs in about 76% co-expression tissues. Although 14 soybean *PHT1* genes more strongly expressed in the roots under the low Pi condition, the response extent were different. Similar subcelluar localizations to the plasma membrane were found amongst most soybean PHT1 proteins. But GmPHT1;8 was not localized to the plasma membrane but to the endoplasmic reticulum, while GmPHT1;10 was localized to Hechtian strands in addition to plasma membranes.

## Methods

### Plant materials

We employed the soybean cultivar (KN18) in all experiments. Plants were grown in a growth chamber under short day conditions (8 hr light/16 hr dark) at a temperature 25°C ~ 28°C. Tissues harvested at two different developmental stages, fully expanded unifoliolate leaf and flowering onset, were evaluated for *GmPHT1* expression patterns and levels in different tissues. We collected pods samples 7, 14 and 21days after flowering. To investigate the effect of external Pi concentrations of *GmPHT1* transcription, plants raised in a hydroponic culture with an initial Pi concentration of 500 μM. Once unifoliolate leaves were fully expanded, the culture solution Pi concentration was changed to 1 μM, 10 μM, 100 μM, 500 μM, 1 mM, 2 mM or 5 mM. Solutions were refreshed once every two days over the course of one week. Each experimental group, containing a minimum of five individual plants per group, was harvested, frozen in liquid nitrogen and stored at -80°C until required. All experiments were repeated three times under the consistent conditions.

### Identification, cloning and expression vector construction of soybean *PHT1* genes

Soybean genome sequences (version 1.09), downloaded from Phytozome V 8.0, and used to obtain a set of known *PHT1* sequences (Additional file 1). Members of the *GmPHT1* family (Additional file 5F) were identified using profile hidden Markov models built by HMMER v3.0 [84], following the HMMER user guide. The *GmPHT1* gene nomenclature is presented in Additional file 5F. A pseudogene (*GmPHT1;15*) was predicted from syntenic analysis using the PGDD or CoGe database.

Given the sequence similarity between the 14 *PHT1* genes in this study, we designed primers specific to the 5^′^ or 3^′^ UTR of each gene (Additional file 5F) for RT-PCR. Subsequently, these sequences were used as templates to clone the CDSs with corresponding primers (Additional file 5E) into an entry vector pGWC [85], next genes were recombined into an appropriate yeast expression vector, pYES-DEST52 (Invitrogen), and plant expression vectors, pEXSG-YFP-GW, with Gateway technology.

### Bioinformatic analysis

To identify the intra-genome (*G. max*) or cross-genome (*G. max* and *M. truncatula*) syntenic relationships we employed, SynMap (http://genomevolution.org/CoGe/SynMap.pl). To investigate the synteny of blocks containing *PHT1* genes, homology data derived from CDS–CDS comparisons made using Blastz, with an E-value cutoff of 1e-5, other parameters were default or recommended. The nine members of the PHT1 gene family was identified (Additional file 5B) in *M. truncatula* as above.

Transmembrane (TM) domains of PHT genes were predicted by TopPred 2 (http://bioweb.pasteur.fr/seqanal/interfaces/toppred.html) [86]. PLACE [31] (http://www.dna.affrc.go.jp/PLACE/signalscan.html) was employed to scan the *cis*-acting elements of the predicted promoter sequences for every *GmPHT1* genes. After alignment of the full *GmPHT1* genes’ CDS by ClustalW (http://www.ebi.ac.uk/Tools/msa/clustalw2/), Ka (non-synonymous substitutions per non-synonymous site) and Ks (synonymous substitutions per synonymous site) of the paralog genes was computed by DnaSP v5 (Additional file 5F) [87].

### Yeast manipulations

The yeast Pi uptake-defective mutant PAM2 (*Δ*pho84*Δ*pho89) [36] was employed to identify the Pi transport activities of the 14 GmPHT1 genes. All GmPHT1 yeast recombinant expression vectors carrying *PHT1* CDS were transformed into PAM2. Transformed cells grew to logarithmic phase in a synthetic liquid medium (SM, 1 liter: 5.9 g YNB (CYN0804, For Medium), 0.77 g mixture of amino acid without Ura (Clontech), 2% raffinose, 6 mM Pi, pH5.8). The cells were harvested when enter the log phase, washed with Pi-free medium, and re-suspended in the same medium to different concentration. For yeast mutant complementation experiments, the yeast cells dilution were plated onto solid, induced or non-induced, medium (1 liter: 5.9 g YNB, 0.77 g mixture of amino acid without Ura, 2% galactose or glucose, 2% agar (#05038, sigma), 10 μM Pi, pH 6.5). Potassium was supplemented with equivalent KCl, and 0.04% bromocresol purple was used as a pH indicator [19], plates were incubated at 30°C, for 3 days. We performed Pi uptake experiments using ^32^Pi as previously reported [20]. Yeast cells grew in liquid, non-induced medium (1 mM Pi) for 6 hr, cells were harvested, and then washed with the Pi-free medium 3 times. Next, yeast cells were grown in liquid, induced medium, without Pi, for 4 hours, harvested and washed with water 3 times. After the final wash, cells were resuspended at 200 mg cells/ml^−1^. Cell suspension 30 μL was added to YNB medium (570 μl), containing 25 mM sodium citrate (pH4.5), 2% glucose and appropriate concentration gradient of Pi (10 μM, 50 μM, 100 μM, 300 μM, 500 μM or 1000 μM). Radioactive ^32^Pi was added to the yeast solution at a final concentration of 0.125 μCi, and cells were incubated at 30°C with gentle agitation for 3 min. Immediately, we added 4 ml of ice-cold stop solution (25 mM sodium citrate buffer, pH 4.5) transferred onto glass fiber filters and washed with an additional 4 ml of stop solution. A scintillation spectroscopy measured the samples radioactivity. All experiments were repeated three times with similar results. The kinetic data was analyzed by nonlinear regression with GraphPad Prism 5 software.

### Total RNA isolation and quantitative reverse transcription-PCR (RT-qPCR)

The procedures used for RNA extraction and cDNA synthesis are as described by Hu, *et al*[88]. All expression experiments were repeated a minimum of three times. The primers for the 15 *PHT1* genes examined by qRT-PCR are listed in Additional file 5F and 14 of the primer pairs had an efficiency greater than 90% as determined by LinRegPCR (http://LinRegPCR.HFRC.nl) [89]. *GmSKIP16*, and *GmUNK1* were used as reference genes for all qRT-PCRs [88]*.* The relative expression was computed following the formula 2^(Cta-Ctb)^, where C_t_a and C_t_b are the average C_t_ values of the reference and target genes, respectively.

### Subcellular localization analysis

Transient expression of YFP tagged GmPHT1 were performed in *Arabidopsis* mesophyll protoplasts through PEG–calcium transfection [90] and in onion epidermal cells by bombardment [20]. Experiments were carried out to analyze subcellular localizations of 14 GmPHT proteins and to investigate the cellular localization of GmPHT1 proteins *in vivo.* Specific subcellular organelles markers, plasma membrane (FM4-64) [91] and endoplasmic reticulum (ER-mRFP) [39], were selected. Section Z-series images were collected at different intervals throughout the specimens to facilitate observation. Twenty to thirty cells were imaged for each experiment. Post-acquisition image analysis and processing was performed using MBF ImageJ, version 1.46.

## Competing interests

The authors declare that they have no competing interests.

## Authors’ contributions

CF carried out all the analysis and interpreted the results, and wrote the manuscript. XW and RH participated in the data mining. YW and CX carried out RT-qPCR. YJ and XZ helped in soybean materials collection and total RNA extraction. YF and CZ conceived the project, supervised the analysis and critically revised the manuscript. All authors read and approved the final manuscript.

## Supplementary Material

Additional file 1 A list of known plant PHT1 genes. Click here for file

Additional file 2 The nomenclature of soybean PHT1s in different papers. Click here for file

Additional file 3 GmPHT1 promoters, genes and gene structures. Click here for file

Additional file 4 PHT1 phylogenetic tree. Click here for file

Additional file 5 **A, The cis-acting regulatory DNA elements present within the 14*GmPHT1*promoters.** B, The *PHT1* gene family in M.truncatula and synteny blocks harbouring *PHT1*s among soybean and Medicago. C, Synteny between *GmPHT1* containing segments in soybean. D, Relative transcript abundance of the various *GmPHT1* genes across different tissues. E, The information of the set of *GmPHT1* genes and the primer sequences used for RT-qPCR and gene cloning experiments. F, The Ks and Ka values among *GmPHT1* genes. Click here for file

Additional file 6 The growth of the yeast double mutant PAM2 in the presence of various concentrations of Pi. Click here for file

Additional file 7 Transient transcription of the GmPHT1;1-YFP, GmPHT1;5-YFP and GmPHT1;6-YFP fusions in plasmolyzed onion epidermal cells. Click here for file

Additional file 8 Motifs and domains shared by the GmPHT1 and other PHT1 proteins. Click here for file

Additional file 9 The appearance of soybean roots in plants subjected to different Pi concentration conditions. Click here for file

## References

[B1] Edger PP Pires JC Gene and genome duplications: the impact of dosage-sensitivity on the fate of nuclear genes Chromosome Res 2009 17 5 699 717 10.1007/s10577-009-9055-9 19802709

[B2] Wang Y Wang X Paterson AH Genome and gene duplications and gene expression divergence: a view from plants Ann N Y Acad Sci 2012 1256 1 14 10.1111/j.1749-6632.2011.06384.x 22257007

[B3] Buggs RJ Elliott NM Zhang L Koh J Viccini LF Soltis DE Soltis PS Tissue-specific silencing of homoeologs in natural populations of the recent allopolyploid Tragopogon mirus New Phytol 2010 186 1 175 183 10.1111/j.1469-8137.2010.03205.x 20409177

[B4] Conant GC Wolfe KH Turning a hobby into a job: how duplicated genes find new functions Nat Rev Genet 2008 9 12 938 950 10.1038/nrg2482 19015656

[B5] Freeling M Bias in plant gene content following different sorts of duplication: tandem, whole-genome, segmental, or by transposition Annu Rev Plant Biol 2009 60 433 453 10.1146/annurev.arplant.043008.092122 19575588

[B6] Cusack BP Wolfe KH When gene marriages don’t work out: divorce by subfunctionalization Trends in genetics: TIG 2007 23 6 270 272 10.1016/j.tig.2007.03.010 17418444

[B7] Blanc G Wolfe KH Functional divergence of duplicated genes formed by polyploidy during Arabidopsis evolution Plant Cell 2004 16 7 1679 1691 10.1105/tpc.021410 15208398 PMC514153

[B8] Moore RC Purugganan MD The evolutionary dynamics of plant duplicate genes Curr Opin Plant Biol 2005 8 2 122 128 10.1016/j.pbi.2004.12.001 15752990

[B9] Schnable JC Springer NM Freeling M Differentiation of the maize subgenomes by genome dominance and both ancient and ongoing gene loss Proc Natl Acad Sci USA 2011 108 10 4069 4074 10.1073/pnas.1101368108 21368132 PMC3053962

[B10] Thomas BC Pedersen B Freeling M Following tetraploidy in an Arabidopsis ancestor, genes were removed preferentially from one homeolog leaving clusters enriched in dose-sensitive genes Genome Res 2006 16 7 934 946 10.1101/gr.4708406 16760422 PMC1484460

[B11] Woodhouse MR Schnable JC Pedersen BS Lyons E Lisch D Subramaniam S Freeling M Following tetraploidy in maize, a short deletion mechanism removed genes preferentially from one of the two homologs PLoS Biol 2010 8 6 e1000409 10.1371/journal.pbio.1000409 20613864 PMC2893956

[B12] Roulin A Auer PL Libault M Schlueter J Farmer A May G Stacey G Doerge RW Jackson SA The fate of duplicated genes in a polyploid plant genome The Plant journal: for cell and molecular biology 2012 73 1 143 153 10.1111/tpj.12026 22974547

[B13] Fernandez A Tzeng YH Hsu SB Subfunctionalization reduces the fitness cost of gene duplication in humans by buffering dosage imbalances BMC Genomics 2011 12 604 10.1186/1471-2164-12-604 22168623 PMC3280233

[B14] Nussaume L Kanno S Javot H Marin E Pochon N Ayadi A Nakanishi TM Thibaud MC Phosphate Import in Plants: Focus on the PHT1 Transporters Front Plant Sci 2011 2 83 22645553 10.3389/fpls.2011.00083 PMC3355772

[B15] Schachtman DP Reid RJ Ayling SM Phosphorus uptake by plants: from soil to cell Plant Physiol 1998 116 2 447 453 10.1104/pp.116.2.447 9490752 PMC1539172

[B16] Nussaume L Kanno S Javot H Marin E Nakanishi TM Thibaud M-C Phosphate import in plants: focus on the PHT1 transporters Frontiers in Plant Science 2011 2 83 22645553 10.3389/fpls.2011.00083 PMC3355772

[B17] Bucher M Functional biology of plant phosphate uptake at root and mycorrhiza interfaces New Phytol 2007 173 1 11 26 10.1111/j.1469-8137.2006.01935.x 17176390

[B18] Lambers H Raven JA Shaver GR Smith SE Plant nutrient-acquisition strategies change with soil age Trends Ecol Evol 2008 23 2 95 103 10.1016/j.tree.2007.10.008 18191280

[B19] Ai P Sun S Zhao J Fan X Xin W Guo Q Yu L Shen Q Wu P Miller AJ Two rice phosphate transporters, OsPht1;2 and OsPht1;6, have different functions and kinetic properties in uptake and translocation Plant J 2009 57 5 798 809 10.1111/j.1365-313X.2008.03726.x 18980647

[B20] Liu J Versaw WK Pumplin N Gomez SK Blaylock LA Harrison MJ Closely related members of the *Medicago truncatula* PHT1 phosphate transporter gene family encode phosphate transporters with distinct biochemical activities J Biol Chem 2008 283 36 24673 24681 10.1074/jbc.M802695200 18596039 PMC3259825

[B21] Lu Q Zhao J Tian J Chen L Sun Z Guo Y Lu X Gu M Xu G Liao H The high-affinity phosphate transporter GmPT5 regulates phosphate transport to nodules and nodulation in soybean Plant Physiol 2012 159 4 1634 1643 10.1104/pp.112.199786 22740613 PMC3425202

[B22] Rae AL Cybinski DH Jarmey JM Smith FW Characterization of two phosphate transporters from barley; evidence for diverse function and kinetic properties among members of the Pht1 family Plant Mol Biol 2003 53 1–2 27 36 14756304 10.1023/B:PLAN.0000009259.75314.15

[B23] Schmutz J Cannon SB Schlueter J Ma J Mitros T Nelson W Hyten DL Song Q Thelen JJ Cheng J Genome sequence of the palaeopolyploid soybean Nature 2010 463 7278 178 183 10.1038/nature08670 20075913

[B24] Schlueter JA Lin JY Schlueter SD Vasylenko-Sanders IF Deshpande S Yi J O’Bleness M Roe BA Nelson RT Scheffler BE Gene duplication and paleopolyploidy in soybean and the implications for whole genome sequencing BMC Genomics 2007 8 330 10.1186/1471-2164-8-330 17880721 PMC2077340

[B25] Severin AJ Cannon SB Graham MM Grant D Shoemaker RC Changes in twelve homoeologous genomic regions in soybean following three rounds of polyploidy Plant Cell 2011 23 9 3129 3136 10.1105/tpc.111.089573 21917551 PMC3203428

[B26] Tamura Y Kobae Y Mizuno T Hata S Identification and expression analysis of arbuscular mycorrhiza-inducible phosphate transporter genes of soybean Biosci Biotechnol Biochem 2012 76 2 309 313 10.1271/bbb.110684 22313769

[B27] Qin L Guo Y Chen L Liang R Gu M Xu G Zhao J Walk T Liao H Functional characterization of 14 pht1 family genes in yeast and their expressions in response to nutrient starvation in soybean PLoS One 2012 7 10 e47726 10.1371/journal.pone.0047726 23133521 PMC3485015

[B28] Wu Z Zhao J Gao R Hu G Gai J Xu G Xing H Molecular cloning, characterization and expression analysis of Two members of the Pht1 family of phosphate transporters in *Glycine max* PLoS One 2011 6 6 e19752 10.1371/journal.pone.0019752 21698287 PMC3115949

[B29] Karandashov V Bucher M Symbiotic phosphate transport in arbuscular mycorrhizas Trends Plant Sci 2005 10 1 22 29 10.1016/j.tplants.2004.12.003 15642520

[B30] Loth-Pereda V Orsini E Courty PE Lota F Kohler A Diss L Blaudez D Chalot M Nehls U Bucher M Structure and expression profile of the phosphate Pht1 transporter gene family in mycorrhizal *Populus trichocarpa* Plant Physiol 2011 156 4 2141 2154 10.1104/pp.111.180646 21705655 PMC3149965

[B31] Higo K Ugawa Y Iwamoto M Korenaga T Plant cis-acting regulatory DNA elements (PLACE) database: 1999 Nucleic Acids Res 1999 27 1 297 300 10.1093/nar/27.1.297 9847208 PMC148163

[B32] Beilstein MA Nagalingum NS Clements MD Manchester SR Mathews S Dated molecular phylogenies indicate a miocene origin for arabidopsis thaliana Proc Natl Acad Sci USA 2010 107 43 18724 18728 10.1073/pnas.0909766107 20921408 PMC2973009

[B33] Young ND Debelle F Oldroyd GED Geurts R Cannon SB Udvardi MK Benedito VA Mayer KFX Gouzy J Schoof H The Medicago genome provides insight into the evolution of rhizobial symbioses Nature 2011 480 7378 520 524 22089132 10.1038/nature10625 PMC3272368

[B34] Subramanian S Kumar S Gene expression intensity shapes evolutionary rates of the proteins encoded by the vertebrate genome Genetics 2004 168 1 373 381 10.1534/genetics.104.028944 15454550 PMC1448110

[B35] Abel S Phosphate sensing in root development Curr Opin Plant Biol 2011 14 3 303 309 10.1016/j.pbi.2011.04.007 21571579

[B36] Martinez P Persson BL Identification, cloning and characterization of a derepressible Na^**+**^**-**coupled phosphate transporter in *Saccharomyces cerevisiae* Mol Gen Genet 1998 258 6 628 638 10.1007/s004380050776 9671031

[B37] Shin H Shin HS Dewbre GR Harrison MJ Phosphate transport in Arabidopsis: Pht1;1 and Pht1;4 play a major role in phosphate acquisition from both low- and high-phosphate environments Plant J 2004 39 4 629 642 10.1111/j.1365-313X.2004.02161.x 15272879

[B38] Bayle V Arrighi J-F Creff A Nespoulous C Vialaret J Rossignol M Gonzalez E Paz-Ares J Nussaume L *Arabidopsis thaliana* high-affinity phosphate transporters exhibit multiple levels of posttranslational regulation Plant Cell 2011 23 4 1523 1535 10.1105/tpc.110.081067 21521698 PMC3101552

[B39] Nelson BK Cai X Nebenfuhr A A multicolored set of in vivo organelle markers for co-localization studies in Arabidopsis and other plants Plant J 2007 51 6 1126 1136 10.1111/j.1365-313X.2007.03212.x 17666025

[B40] Smith FW Mudge SR Rae AL Glassop D Phosphate transport in plants Plant Soil 2003 248 1 71 83

[B41] Raghothama KG Phosphate transport and signaling Curr Opin Plant Biol 2000 3 3 182 187 10837272

[B42] Miura K Lee J Gong Q Ma S Jin JB Yoo CY Miura T Sato A Bohnert HJ Hasegawa PM SIZ1 regulation of phosphate starvation-induced root architecture remodeling involves the control of auxin accumulation Plant Physiol 2011 155 2 1000 1012 10.1104/pp.110.165191 21156857 PMC3032448

[B43] Svistoonoff S Creff A Reymond M Sigoillot-Claude C Ricaud L Blanchet A Nussaume L Desnos T Root tip contact with low-phosphate media reprograms plant root architecture Nat Genet 2007 39 6 792 796 10.1038/ng2041 17496893

[B44] Lopez-Bucio J Cruz-Ramirez A Herrera-Estrella L The role of nutrient availability in regulating root architecture Curr Opin Plant Biol 2003 6 3 280 287 10.1016/S1369-5266(03)00035-9 12753979

[B45] Karandashov V Nagy R Wegmuller S Amrhein N Bucher M Evolutionary conservation of a phosphate transporter in the arbuscular mycorrhizal symbiosis Proc Natl Acad Sci USA 2004 101 16 6285 6290 10.1073/pnas.0306074101 15075387 PMC395961

[B46] Wellman CH Osterloff PL Mohiuddin U Fragments of the earliest land plants Nature 2003 425 6955 282 285 10.1038/nature01884 13679913

[B47] Redecker D Kodner R Graham LE Glomalean fungi from the Ordovician Science 2000 289 5486 1920 1921 10.1126/science.289.5486.1920 10988069

[B48] Bonfante P Genre A Mechanisms underlying beneficial plant-fungus interactions in mycorrhizal symbiosis Nat Commun 2010 1 48 20975705 10.1038/ncomms1046

[B49] Adams KL Wendel JF Polyploidy and genome evolution in plants Curr Opin Plant Biol 2005 8 2 135 141 10.1016/j.pbi.2005.01.001 15752992

[B50] Tang H Bowers JE Wang X Ming R Alam M Paterson AH Synteny and collinearity in plant genomes Science 2008 320 5875 486 488 10.1126/science.1153917 18436778

[B51] Blanc G Hokamp K Wolfe KH A recent polyploidy superimposed on older large-scale duplications in the Arabidopsis genome Genome Res 2003 13 2 137 144 10.1101/gr.751803 12566392 PMC420368

[B52] Mudge SR Rae AL Diatloff E Smith FW Expression analysis suggests novel roles for members of the Pht1 family of phosphate transporters in Arabidopsis Plant J 2002 31 3 341 353 10.1046/j.1365-313X.2002.01356.x 12164813

[B53] Okumura S Mitsukawa N Shirano Y Shibata D Phosphate transporter gene family of *Arabidopsis thaliana* DNA Res 1998 5 5 261 269 10.1093/dnares/5.5.261 9872450

[B54] Tuskan GA Difazio S Jansson S Bohlmann J Grigoriev I Hellsten U Putnam N Ralph S Rombauts S Salamov A The genome of black cottonwood, *Populus trichocarpa* (Torr. & Gray) Science 2006 313 5793 1596 1604 10.1126/science.1128691 16973872

[B55] Guyot R Keller B Ancestral genome duplication in rice Genome / National Research Council Canada = Genome / Conseil national de recherches Canada 2004 47 3 610 614 10.1139/g04-016 15190378

[B56] Yang SY Gronlund M Jakobsen I Grotemeyer MS Rentsch D Miyao A Hirochika H Kumar CS Sundaresan V Salamin N Nonredundant Regulation of Rice Arbuscular Mycorrhizal Symbiosis by Two Members of the PHOSPHATE TRANSPORTER1 Gene Family Plant Cell 2012 24 10 4236 4251 10.1105/tpc.112.104901 23073651 PMC3517247

[B57] Paszkowski U Kroken S Roux C Briggs SP Rice phosphate transporters include an evolutionarily divergent gene specifically activated in arbuscular mycorrhizal symbiosis Proc Natl Acad Sci USA 2002 99 20 13324 13329 10.1073/pnas.202474599 12271140 PMC130632

[B58] Hurles M Gene duplication: the genomic trade in spare parts PLoS Biol 2004 2 7 E206 10.1371/journal.pbio.0020206 15252449 PMC449868

[B59] Nagarajan VK Jain A Poling MD Lewis AJ Raghothama KG Smith AP Arabidopsis Pht1;5 Mobilizes Phosphate between Source and Sink Organs and Influences the Interaction between Phosphate Homeostasis and Ethylene Signaling Plant Physiol 2011 156 3 1149 1163 10.1104/pp.111.174805 21628630 PMC3135966

[B60] Pumplin N Zhang X Noar RD Harrison MJ Polar localization of a symbiosis-specific phosphate transporter is mediated by a transient reorientation of secretion Proc Natl Acad Sci 2012 109 11 E665 E672 22355114 10.1073/pnas.1110215109 PMC3306687

[B61] Harrison MJ Dewbre GR Liu J A phosphate transporter from *Medicago truncatula* involved in the acquisition of phosphate released by arbuscular mycorrhizal fungi Plant Cell 2002 14 10 2413 2429 10.1105/tpc.004861 12368495 PMC151226

[B62] Kobae Y Hata S Dynamics of periarbuscular membranes visualized with a fluorescent phosphate transporter in arbuscular mycorrhizal roots of rice Plant Cell Physiol 2010 51 3 341 353 10.1093/pcp/pcq013 20097910

[B63] Javot H Pumplin N Harrison MJ Phosphate in the arbuscular mycorrhizal symbiosis: transport properties and regulatory roles Plant Cell Environ 2007 30 3 310 322 10.1111/j.1365-3040.2006.01617.x 17263776

[B64] Nagy R Karandashov V Chague V Kalinkevich K Tamasloukht M Xu G Jakobsen I Levy AA Amrhein N Bucher M The characterization of novel mycorrhiza-specific phosphate transporters from *Lycopersicon esculentum* and *Solanum tuberosum* uncovers functional redundancy in symbiotic phosphate transport in solanaceous species Plant J 2005 42 2 236 250 10.1111/j.1365-313X.2005.02364.x 15807785

[B65] Huang C Barker SJ Langridge P Smith FW Graham RD Zinc deficiency up-regulates expression of high-affinity phosphate transporter genes in both phosphate-sufficient and -deficient barley roots Plant Physiol 2000 124 1 415 422 10.1104/pp.124.1.415 10982454 PMC59154

[B66] DeLuna A Vetsigian K Shoresh N Hegreness M Colon-Gonzalez M Chao S Kishony R Exposing the fitness contribution of duplicated genes Nat Genet 2008 40 5 676 681 10.1038/ng.123 18408719

[B67] Gu Z Steinmetz LM Gu X Scharfe C Davis RW Li WH Role of duplicate genes in genetic robustness against null mutations Nature 2003 421 6918 63 66 10.1038/nature01198 12511954

[B68] Henderson PJ The 12-transmembrane helix transporters Curr Opin Cell Biol 1993 5 4 708 721 10.1016/0955-0674(93)90144-F 8257611

[B69] Petersson J Pattison J Kruckeberg AL Berden JA Persson BL Intracellular localization of an active green fluorescent protein-tagged Pho84 phosphate permease in *Saccharomyces cerevisiae* FEBS Lett 1999 462 1–2 37 42 10580087 10.1016/s0014-5793(99)01471-4

[B70] Lau W-TW Howson RW Malkus P Schekman R O’Shea EK Pho86p, an endoplasmic reticulum (ER) resident protein in *Saccharomyces cerevisiae*, is required for ER exit of the high-affinity phosphate transporter Pho84p Proc Natl Acad Sci 2000 97 3 1107 1112 10.1073/pnas.97.3.1107 10655492 PMC15537

[B71] Raghothama KG Phosphate Acquisition Annu Rev Plant Physiol Plant Mol Biol 1999 50 665 693 10.1146/annurev.arplant.50.1.665 15012223

[B72] Zhao J Fu J Liao H He Y Nian H Hu Y Qiu L Dong Y Yan X Characterization of root architecture in an applied core collection for phosphorus efficiency of soybean germplasm Chinese Science Bulletin 2004 49 15 1611 1620

[B73] Williamson LC Ribrioux SP Fitter AH Leyser HM Phosphate availability regulates root system architecture in Arabidopsis Plant Physiol 2001 126 2 875 882 10.1104/pp.126.2.875 11402214 PMC111176

[B74] Perez-Torres CA Lopez-Bucio J Cruz-Ramirez A Ibarra-Laclette E Dharmasiri S Estelle M Herrera-Estrella L Phosphate availability alters lateral root development in Arabidopsis by modulating auxin sensitivity via a mechanism involving the TIR1 auxin receptor Plant Cell 2008 20 12 3258 3272 10.1105/tpc.108.058719 19106375 PMC2630440

[B75] Yang SY Paszkowski U Phosphate import at the arbuscule: just a nutrient? Mol Plant Microbe Interact 2011 24 11 1296 1299 10.1094/MPMI-06-11-0151 21995797

[B76] Daram P Brunner S Rausch C Steiner C Amrhein N Bucher M Pht2;1 encodes a low-affinity phosphate transporter from Arabidopsis Plant Cell 1999 11 11 2153 2166 10559441 10.1105/tpc.11.11.2153 PMC144119

[B77] Nagy R Vasconcelos MJV Zhao S McElver J Bruce W Amrhein N Raghothama KG Bucher M Differential Regulation of Five *Pht1* Phosphate Transporters from Maize (*Zea mays* L.) Plant Biology 2006 8 2 186 197 10.1055/s-2005-873052 16547863

[B78] Jia H Ren H Gu M Zhao J Sun S Zhang X Chen J Wu P Xu G The Phosphate Transporter Gene OsPht1;8 Is Involved in Phosphate Homeostasis in Rice Plant Physiol 2011 156 3 1164 1175 10.1104/pp.111.175240 21502185 PMC3135946

[B79] Chapin LJ Jones ML Ethylene regulates phosphorus remobilization and expression of a phosphate transporter (PhPT1) during petunia corolla senescence J Exp Bot 2009 60 7 2179 2190 10.1093/jxb/erp092 19380421 PMC2682506

[B80] Lauer MJ Blevins DG Sierzputowska-Gracz H ^31^P-nuclear magnetic resonance determination of phosphate compartmentation in leaves of reproductive soybeans (*Glycine max* L.) as affected by phosphate nutrition Plant Physiol 1989 89 4 1331 10.1104/pp.89.4.1331 16666705 PMC1056017

[B81] Lin WY Lin SI Chiou TJ Molecular regulators of phosphate homeostasis in plants J Exp Bot 2009 60 5 1427 1438 10.1093/jxb/ern303 19168668

[B82] Nick P Signals, motors, morphogenesis—the cytoskeleton in plant development Plant Biology 1999 1 169 179 10.1111/j.1438-8677.1999.tb00240.x

[B83] Mravec J Skupa P Bailly A Hoyerova K Krecek P Bielach A Petrasek J Zhang J Gaykova V Stierhof YD Subcellular homeostasis of phytohormone auxin is mediated by the ER-localized PIN5 transporter Nature 2009 459 7250 1136 1140 10.1038/nature08066 19506555

[B84] Finn RD Clements J Eddy SR HMMER web server: interactive sequence similarity searching Nucleic Acids Res 2011 39 suppl 2 W29 W37 21593126 10.1093/nar/gkr367 PMC3125773

[B85] Chen Q-J Zhou H-M Chen J Wang X-C Using a modified TA cloning method to create entry clones Anal Biochem 2006 358 1 120 125 10.1016/j.ab.2006.08.015 16970900

[B86] von Heijne G Membrane protein structure prediction. Hydrophobicity analysis and the positive-inside rule J Mol Biol 1992 225 2 487 494 10.1016/0022-2836(92)90934-C 1593632

[B87] Librado P Rozas J DnaSP v5: a software for comprehensive analysis of DNA polymorphism data Bioinformatics 2009 25 11 1451 1452 10.1093/bioinformatics/btp187 19346325

[B88] Hu R Fan C Li H Zhang Q Fu Y-F Evaluation of putative reference genes for gene expression normalization in soybean by quantitative real-time RT-PCR BMC Mol Biol 2009 10 1 93 10.1186/1471-2199-10-93 19785741 PMC2761916

[B89] Ruijter JM Ramakers C Hoogaars WM Karlen Y Bakker O van den Hoff MJ Moorman AF Amplification efficiency: linking baseline and bias in the analysis of quantitative PCR data Nucleic Acids Res 2009 37 6 e45 10.1093/nar/gkp045 19237396 PMC2665230

[B90] Yoo S-D Cho Y-H Sheen J Arabidopsis mesophyll protoplasts: a versatile cell system for transient gene expression analysis Nat Protocols 2007 2 7 1565 1572 10.1038/nprot.2007.199 17585298

[B91] Bolte S Talbot C Boutte Y Catrice O Read N Satiat-Jeunemaitre B FM-dyes as experimental probes for dissecting vesicle trafficking in living plant cells J Microsc 2004 214 2 159 173 10.1111/j.0022-2720.2004.01348.x 15102063

