# Supplementary material for: The pattern of Phosphate transporter 1 genes evolutionary divergence in Glycine max L
Source: BMC Plant Biol. 2013 Mar 20;13:48. doi: 10.1186/1471-2229-13-48 (PMC3621523; doi:10.1186/1471-2229-13-48)
Supplement: Additional file 1 — A list of known plant PHT1 genes. [file 1471-2229-13-48-S1.pdf]

Additional file 1 A list of known plant *PHT1* genes.

Species	Gene name	Accession	References
Glomus versiforme	GvPT▽	Q00908	[1]
Piriformospra indica	PiPT	A8N031	[2]
Neurospora crassa	PHO5▽	Q01395	[3]
	PHO4▽	P15710	[4]
Saccharomyces cerevisiae	PHO84▽	P25297	[5]
	PHO89▽	P38361	[6]
Lycopersicon esculentum	LePT1▽	O24029	[7]
	LePT2	O22549	[8]
	LePT3	Q5MJA7	[9]
	LePT4*△	Q563I3	[10]
	LePT5	Q563I2	
Solanum tuberosum	StPT1▽	Q43650	[11]
	StPT2▽	Q41479	
	StPT3*▽	Q8W4W9	[12]
	StPT4△	Q5ICC1	[13]
	StPT5	Q563I1	[10]
Nicotiana tabacum	NtPT1	Q9ST22	[14]
	NtPT2	Q9AYT3	
	NtPT3	Q9AYT2	
	NtPT3	A0T3D8	[15]
	NtPT4	A0T3E1	
	NtPT5	A0T3E4	
Solanum melongena	SmPT1	A0T3D3	
	SmPT2	A0T3D5	
	SmPT3	A0T3D7	
	SmPT4	A0T3E0	

	SmPT5	A0T3E3	
Capsicum frutescens	CfPT1	A0T3D2	
	CfPT2	A0T3D4	
	CfPT3	A0T3D6	
	CfPT4	A0T3D9	
	CfPT5	A0T3E2	
Lotus japonicas	LjPT1	Q1T6Z7	[16]
	LjPT2	Q1T6Z6	
	LjPT3*	Q1T6Z8	
Catharanthus roseus	CrPT1	O22055	[17]
Medicago truncatula	MtPT1	O22301	[18]
	MtPT2▽	O22302	
	MtPT3△	A5H2U5	[19]
	MtPT4*△	Q8GSG4	[20]
	MtPT5▽	A5H2U6	[19]
	MtPT6	B2G3Q0	
Lupinus albus	LaPT1	Q9ARI9	[21]
	LaPT2	Q9AU00	
Sesbania rostrata	SrPT1	Q9AVR0	
Triticum aestivum	TaPht1*	Q5CC72	[22]
	TaPht2	Q93WR0	[23]
	TaPht8	Q7YMD9	
Triticum monococcum	TmPT1	Q5NKQ7	
Zea mays	ZmPHT1	Q49B46, GRMZM2G154090	[24]
	ZmPHT2	Q49B45, GRMZM2G024935	

	ZmPHT3	Q49B44,<br>GRMZM2G112377	
	ZmPHT4	Q49B43,<br>GRMZM2G024935	
	ZmPHT5	Q49B42,<br>GRMZM2G041595	
	ZmPHT6*	Q5CC71,<br>GRMZM2G181102	[22]
Hordeum vulgare	HvPht1;1▽	Q8H6E0	[25]
	HvPT2	Q84LH9	
	HvPT3	Q84VI5	
	HvPT4	Q84JQ1	
	HvPT5	Q84VI7	
	HvPht1;6△	Q8H6D9	[25]
	HvPT7	Q84VI6	
	HvPT8*	Q6Y3A2	[26]
	HvPT9	Q8H6E0	
Petunia hybrida	*PhPT1*	B2CPI3	[27]
	PhPT2	B2CPI4	
	*PhPT3**	B2CPI5	
	*PhPT4**	B2CPI6	
	*PhPT5**	B2CPI7	
Arabidopsis thaliana	*AtPHT1;1*▽	At5g43350	[28-30]
	AtPHT1;2	At5g43370	
	AtPHT1;3	At5g43360	
	AtPHT1;4▽	At2g38940	
	AtPHT1;5	At2g32830	
	AtPHT1;6	At5g43340	
	AtPHT1;7	At3g54700	

	AtPHT1;8	At1g20860	
	AtPHT1;9	At1g76430	
Oryza sativa	*OsPT1*	Os03g05620	[31]
	OsPT2△	Os03g05640	
	OsPT3	Os10g30770	
	OsPT4	Os04g10750	
	OsPT5	Os04g10690	
	OsPT6▽	Os08g45000	
	OsPT7	Os03g04360	
	OsPT8▽	Os10g30790	
	OsPT9	Os06g21950	
	OsPT10	Os06g21950	
	*OsPT11**	Os01g46860	
	OsPT12	Os03g05610	
	OsPT13	Os04g10800	
Populus trichocarpa	*PtPHT1;1*	POPTR_0010s08270	[32]
	PtPHT1;2	POPTR_0010s08250	
	PtPHT1;3	POPTR_0010s08230	
	PtPHT1;4	POPTR_0005s24490	
	PtPHT1;5	POPTR_0002s04000	
	PtPHT1;6	POPTR_0005s15880	
	PtPHT1;7	POPTR_0005s24500	
	*PtPHT1;8**	POPTR_0019s08990	
	PtPHT1;9	POPTR_0002s00700	
	*PtPHT1;10**	POPTR_0015s06110	
	PtPHT1;11	POPTR_0005s27740	
	PtPHT1;12	POPTR_0001s32590	

Note: *, its expression induced by mycorrhizal fungi; ▽, proteins with Pi high-affinity activity.

△, proteins with Pi low-affinity activity.

References:

1. Harrison MJ, van Buuren ML: **A phosphate transporter from the mycorrhizal fungus *Glomus versiforme***. *Nature* 1995, **378**(6557):626-629.
2. Yadav V, Kumar M, Deep DK, Kumar H, Sharma R, Tripathi T, Tuteja N, Saxena AK, Johri AK: **A phosphate transporter from the root endophytic fungus Piriformospora indica plays a role in the phosphate transport to the host plant**. *J Biol Chem* 2010.
3. Versaw WK, Metzenberg RL: **Repressible cation-phosphate symporters in *Neurospora crassa***. *Proc Natl Acad Sci U S A* 1995, **92**(9):3884-3887.
4. Mann BJ, Bowman BJ, Grotelueschen J, Metzenberg RL: **Nucleotide sequence of pho-4+, encoding a phosphate-repressible phosphate permease ofNeurospora crassa**. *Gene* 1989, **83**(2):281-289.
5. Bun-Ya M, Nishimura M, Harashima S, Oshima Y: **The PHO84 gene of Saccharomyces cerevisiae encodes an inorganic phosphate transporter**. *Mol Cell Biol* 1991, **11**(6):3229-3238.
6. Martinez P, Persson BL: **Identification, cloning and characterization of a derepressible Na+-coupled phosphate transporter in *Saccharomyces cerevisiae***. *Mol Gen Genet* 1998, **258**(6):628-638.
7. Daram P, Brunner S, Persson BL, Amrhein N, Bucher M: **Functional analysis and cell-specific expression of a phosphate transporter from tomato**. *Planta* 1998, **206**(2):225-233.
8. Liu C, Muchhal US, Uthappa M, Kononowicz AK, Raghothama KG: **Tomato phosphate transporter genes are differentially regulated in plant tissues by phosphorus**. *Plant Physiol* 1998, **116**(1):91-99.
9. Xu GH, Chague V, Melamed-Bessudo C, Kapulnik Y, Jain A, Raghothama KG, Levy AA, Silber A: **Functional characterization of LePT4: a phosphate transporter in tomato with mycorrhiza-enhanced expression**. *J Exp Bot* 2007, **58**(10):2491-2501.
10. Nagy R, Karandashov V, Chague V, Kalinkevich K, Tamasloukht M, Xu G, Jakobsen I, Levy AA, Amrhein N, Bucher M: **The characterization of novel mycorrhiza-specific phosphate transporters from *Lycopersicon esculentum* and *Solanum tuberosum* uncovers functional redundancy in symbiotic phosphate transport in solanaceous species**. *Plant J* 2005, **42**(2):236-250.
11. Leggewie G, Willmitzer L, Riesmeier JW: **Two cDNAs from Potato Are Able to Complement a Phosphate Uptake-Deficient Yeast Mutant: Identification of Phosphate Transporters from Higher Plants**. *Plant Cell* 1997, **9**(3):381-392.
12. Rausch C, Daram P, Brunner S, Jansa J, Laloi M, Leggewie G, Amrhein N, Bucher M: **A phosphate transporter expressed in arbuscule-containing cells in potato**. *Nature* 2001, **414**(6862):462-470.
13. Karandashov V, Bucher M: **Symbiotic phosphate transport in arbuscular mycorrhizas**. *Trends Plant Sci* 2005, **10**(1):22-29.
14. Kai M, Takazumi K, Adachi H, Wasaki J, Shinano T, Osaki M: **Cloning and characterization of four phosphate transporter cDNAs in tobacco**. *Plant Science* 2002, **163**(4):837-846.
15. Chen A, Hu J, Sun S, Xu G: **Conservation and divergence of both phosphate- and mycorrhiza-regulated physiological responses and expression patterns of phosphate transporters in solanaceous species**. *New Phytol* 2007, **173**(4):817-831.
16. Maeda D, Ashida K, Iguchi K, Chechetka SA, Hijikata A, Okusako Y, Deguchi Y, Izui K, Hata S:

Knockdown of an arbuscular mycorrhiza-inducible phosphate transporter gene of *Lotus japonicus* suppresses mutualistic symbiosis. *Plant Cell Physiol* 2006, **47**(7):807-817.
17. Kai M, Masuda Y, Kikuchi Y, Osaki M, Tadano T: **Isolation and characterization of a cDNA from *Catharanthus roseus* which is highly homologous with phosphate transporter**. *Soil science and plant nutrition* 1997, **43**(1):227-235.
18. Liu H, Trieu AT, Blaylock LA, Harrison MJ: **Cloning and characterization of two phosphate transporters from *Medicago truncatula* roots: regulation in response to phosphate and to colonization by arbuscular mycorrhizal (AM) fungi**. *Mol Plant Microbe Interact* 1998, **11**(1):14-22.
19. Liu J, Versaw WK, Pumplin N, Gomez SK, Blaylock LA, Harrison MJ: **Closely related members of the *Medicago truncatula* PHT1 phosphate transporter gene family encode phosphate transporters with distinct biochemical activities**. *J Biol Chem* 2008, **283**(36):24673-24681.
20. Harrison MJ, Dewbre GR, Liu J: **A phosphate transporter from *Medicago truncatula* involved in the acquisition of phosphate released by arbuscular mycorrhizal fungi**. *Plant Cell* 2002, **14**(10):2413-2429.
21. Liu J, Uhde-Stone C, Li A, Vance C, Allan D: **A phosphate transporter with enhanced expression in proteoid roots of white lupin (*Lupinus albus* L.)**. *Plant and Soil* 2001, **237**(2):257-266.
22. Glassop D, Smith S, Smith F: **Cereal phosphate transporters associated with the mycorrhizal pathway of phosphate uptake into roots**. *Planta* 2005, **222**(4):688-698.
23. Davies TGE, Ying J, Xu Q, Li ZS, Li J, Gordon-Weeks R: **Expression analysis of putative high-affinity phosphate transporters in Chinese winter wheats**. *Plant, Cell & Environment* 2002, **25**(10):1325-1339.
24. Nagy R, Vasconcelos MJV, Zhao S, McElver J, Bruce W, Amrhein N, Raghothama KG, Bucher M: **Differential Regulation of Five Pht1 Phosphate Transporters from Maize (Zea mays L.)**. *Plant Biol (Stuttg)* 2006, **8**(02):186,197.
25. Rae AL, Cybinski DH, Jarmey JM, Smith FW: **Characterization of two phosphate transporters from barley; evidence for diverse function and kinetic properties among members of the Pht1 family**. *Plant Mol Biol* 2003, **53**(1-2):27-36.
26. Grace EJ, Cotsaftis O, Tester M, Smith FA, Smith SE: **Arbuscular mycorrhizal inhibition of growth in barley cannot be attributed to extent of colonization, fungal phosphorus uptake or effects on expression of plant phosphate transporter genes**. *New Phytol* 2008.
27. Wegmuller S, Svistoonoff S, Reinhardt D, Stuurman J, Amrhein N, Bucher M: **A transgenic dTph1 insertional mutagenesis system for forward genetics in mycorrhizal phosphate transport of Petunia**. *Plant J* 2008, **54**(6):1115-1127.
28. Muchhal US, Pardo JM, Raghothama KG: **Phosphate transporters from the higher plant *Arabidopsis thaliana***. *Proc Natl Acad Sci U S A* 1996, **93**(19):10519-10523.
29. Mudge SR, Rae AL, Diatloff E, Smith FW: **Expression analysis suggests novel roles for members of the Pht1 family of phosphate transporters in Arabidopsis**. *Plant J* 2002, **31**(3):341-353.
30. Okumura S, Mitsukawa N, Shirano Y, Shibata D: **Phosphate transporter gene family of *Arabidopsis thaliana***. *DNA Res* 1998, **5**(5):261-269.
31. Paszkowski U, Kroken S, Roux C, Briggs SP: **Rice phosphate transporters include an evolutionarily divergent gene specifically activated in arbuscular mycorrhizal symbiosis**.

Proc Natl Acad Sci U S A 2002, **99**(20):13324-13329.

32. Loth-Pereda V, Orsini E, Courty PE, Lota F, Kohler A, Diss L, Blaudez D, Chalot M, Nehls U, Bucher M *et al*: **Structure and expression profile of the phosphate Pht1 transporter gene family in mycorrhizal *Populus trichocarpa***. *Plant Physiol* 2011.
