# Supplementary material for: The pattern of Phosphate transporter 1 genes evolutionary divergence in Glycine max L
Source: BMC Plant Biol. 2013 Mar 20;13:48. doi: 10.1186/1471-2229-13-48 (PMC3621523; doi:10.1186/1471-2229-13-48)
Supplement: Additional file 2 — The nomenclature of soybean PHT1s in different papers. [file 1471-2229-13-48-S2.pdf]

Additional file 2 The nomenclature of soybean *PHT1*s in different papers

**Gene Identifier**	***GmPHT1* (This study)**	***GmPT* (Lu et al. [1])**	***GmPT* (Tamura et al. [2])**	***GmPT* (Wu et al. [3])**
Glyma10g33030	*GmPHT1;1*	*GmPT7*	*GmPT6*	*GmPT1*
Glyma03g31950	*GmPHT1;2*	*GmPT2*	*GmPT4*	---
Glyma10g00950	*GmPHT1;3*	*GmPT4*	*GmPT13*	---
Glyma10g04230	*GmPHT1;4*	*GmPT5*	*GmPT5*	---
Glyma20g34610	*GmPHT1;5*	*GmPT13*	*GmPT2*	*GmPT2*
Glyma10g33020	*GmPHT1;6*	*GmPT6*	*GmPT1*	---
Glyma19g34710	*GmPHT1;7*	*GmPT11*	*GmPT12*	---
Glyma20g02660	*GmPHT1;8*	*GmPT12*	*GmPT8*	---
Glyma07g34870	*GmPHT1;9*	*GmPT3*	*GmPT9*	---
Glyma20g34620	*GmPHT1;10*	*GmPT14*	*GmPT14*	---
Glyma14g36650	*GmPHT1;11*	*GmPT10*	*GmPT7*	---
Glyma14g28780	*GmPHT1;12*	*GmPT9*	*GmPT11*	---
Glyma13g08720	*GmPHT1;13*	*GmPT8*	*GmPT10*	---
Glyma02g00840	*GmPHT1;14*	*GmPT1*	*GmPT3*	---
Glyma13g18420	*GmPHT1;15*	---	---	---

'---' indicates genes Not-identified in the paper. The same color background indicates paralogous pair genes.

References:

1. Lu Q, Zhao J, Tian J, Chen L, Sun Z, Guo Y, Lu X, Gu M, Xu G, Liao H: **The high-affinity phosphate transporter GmPT5 regulates phosphate transport to nodules and nodulation in soybean**. *Plant Physiol* 2012.
2. Tamura Y, Kobae Y, Mizuno T, Hata S: **Identification and expression analysis of arbuscular mycorrhiza-inducible phosphate transporter genes of soybean**. *Biosci Biotechnol Biochem* 2012, **76**(2):309-313.

3. Wu Z, Zhao J, Gao R, Hu G, Gai J, Xu G, Xing H: **Molecular Cloning, Characterization and Expression Analysis of Two Members of the Pht1 Family of Phosphate Transporters in *Glycine max***. *PLoS One* 2011, **6**(6):e19752.
