# Supplementary material for: The pattern of Phosphate transporter 1 genes evolutionary divergence in Glycine max L
Source: BMC Plant Biol. 2013 Mar 20;13:48. doi: 10.1186/1471-2229-13-48 (PMC3621523; doi:10.1186/1471-2229-13-48)
Supplement: Additional file 3 — GmPHT1 promoters, genes and gene structures. [file 1471-2229-13-48-S3.pdf]

A

	GmPHT1;2	*GmPHT1;7*	*GmPHT1;4*	*GmPHT1;1*	*GmPHT1;5*	*GmPHT1;6*	*GmPHT1;10*	*GmPHT1;3*	*GmPHT1;14*	*GmPHT1;11*	*GmPHT1;12*	*GmPHT1;13*	*GmPHT1;8*	*GmPHT1;9*
GmPHT1;2		**96.2**	82.9	71	70.9	76.4	76.1	72.1	71.6	60.6	60.8	59.3	58.8	58.7
GmPHT1;7	**64.2**		83.2	71.3	71.1	75.9	75.6	72.4	72.2	59.9	60.7	59.3	58.7	58.6
GmPHT1;4	36.7	37.6		70.3	70.2	74.6	74.7	71.9	71.5	62.4	61.6	61.3	57.9	57.9
GmPHT1;1	35.2	35.1	34.5		**95.8**	74.1	74.3	83.1	83	59.9	60.8	60.3	57.4	57.3
GmPHT1;5	34.2	34.8	35.3	**70.2**		73.3	73.5	83	82.8	59.6	60.7	60.2	56.8	56.8
GmPHT1;6	42.6	42.8	34.6	35.7	36.7		**96.9**	73.7	73.8	62.8	61.1	60.8	58.7	58.7
GmPHT1;10	42.9	44.1	35.8	36.8	37.5	**60.8**		73.8	73.7	62.7	61.5	61.2	59.1	58.7
GmPHT1;3	37.7	38.2	35.8	37.7	37.8	36.1	37.4		**95.7**	62.1	61.4	60.3	58.8	59
GmPHT1;14	35.4	36.0	34.2	35.5	35.7	34.4	33.2	**43.1**		61.9	61.8	60.1	59.1	59.4
GmPHT1;11	38.0	37.8	35.2	36.1	35.4	37.4	38.3	37.3	34.8		58.8	59.5	56.1	56.4
GmPHT1;12	34.4	36.5	34.4	42.1	40.8	35.9	37.7	35.9	35.3	34.5		**90.1**	55.5	56.7
GmPHT1;13	35.6	36.7	34.2	41.5	41.5	37.0	38.4	36.2	34.5	34.2	**63.5**		54.9	55.5
GmPHT1;8	35.6	35.6	33.3	35.8	35.9	36.8	35.8	34.7	33.0	34.9	36.9	36.9		**94.6**
GmPHT1;9	34.0	33.4	31.4	34.6	35.2	33.8	33.7	35.3	32.7	34.6	34.7	34.7	**46.9**	

B

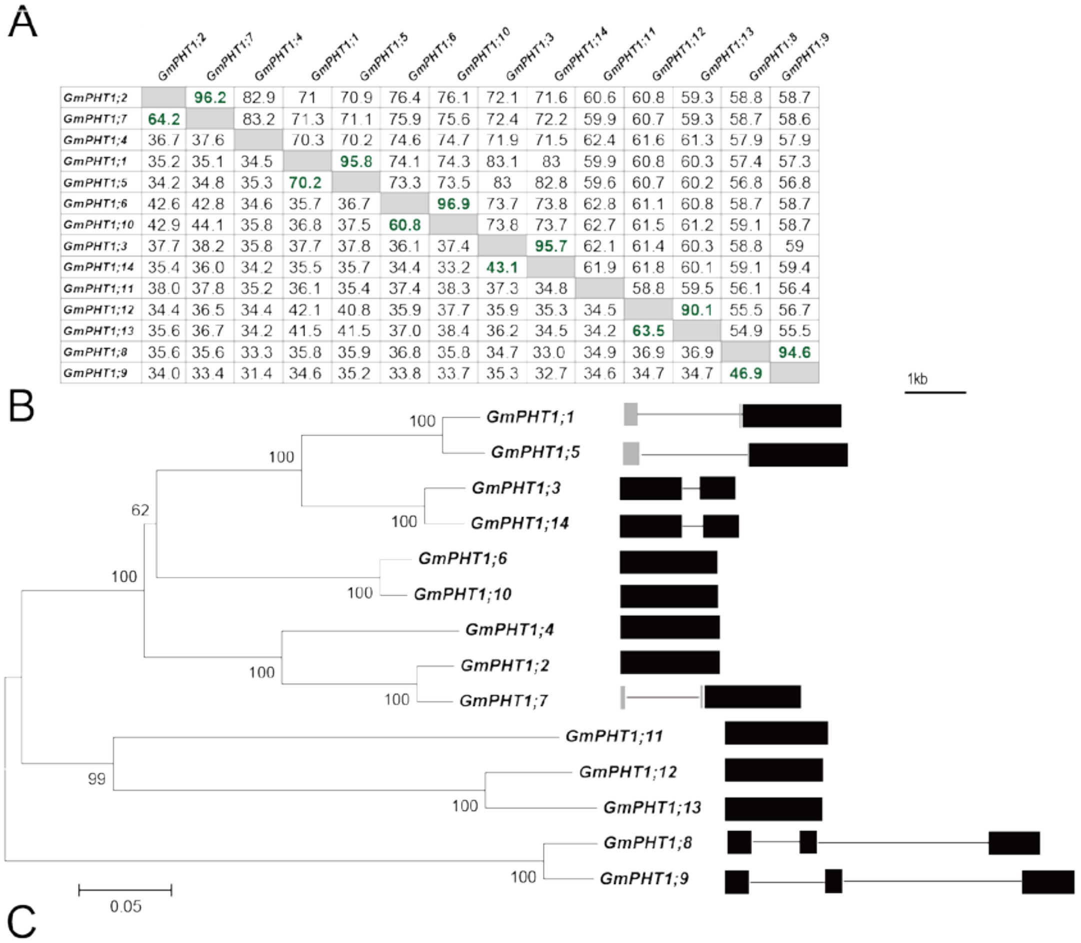

C

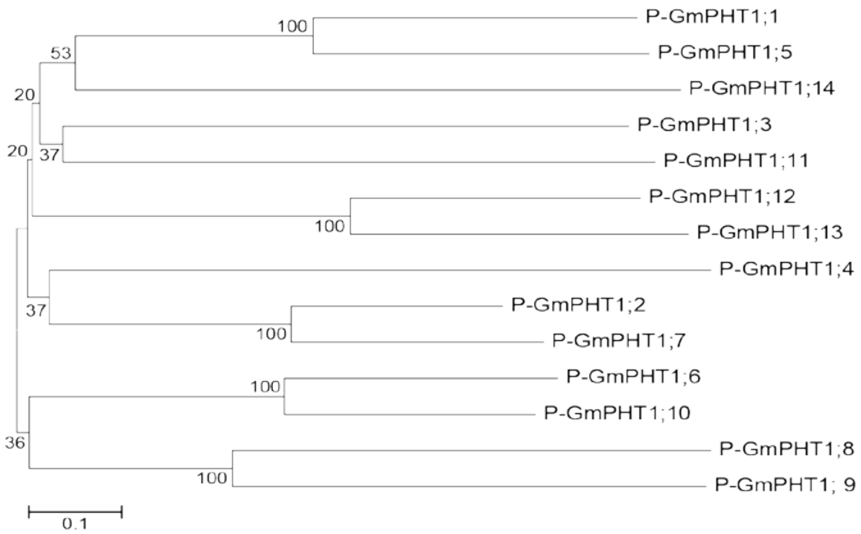

Additional file 3. *GmPHT1* promoters, genes and gene structures. A, Sequence similarity between individual *GmPHT1* genes (upper triangle) and their promoters (lower triangle); The similarity between paralogous pairs shown in green. B, Phylogenetic tree of the 14 *GmPHT1* genes based on their DNA sequence. The black numbers shown are bootstrap values, and the

green ones similarity. The structure of the 14 *GmPHT1*s (right) was derived from the genome annotation. Open boxes indicate the CDSs, black lines introns, and red boxes introns in the 5’-UTR. C, Phylogenetic tree of the *GmPHT1* promoters. The black numbers shown are bootstrap values, and the green ones similarity.
