# Supplementary material for: The pattern of Phosphate transporter 1 genes evolutionary divergence in Glycine max L
Source: BMC Plant Biol. 2013 Mar 20;13:48. doi: 10.1186/1471-2229-13-48 (PMC3621523; doi:10.1186/1471-2229-13-48)
Supplement: Additional file 4 — PHT1 phylogenetic tree. [file 1471-2229-13-48-S4.pdf]

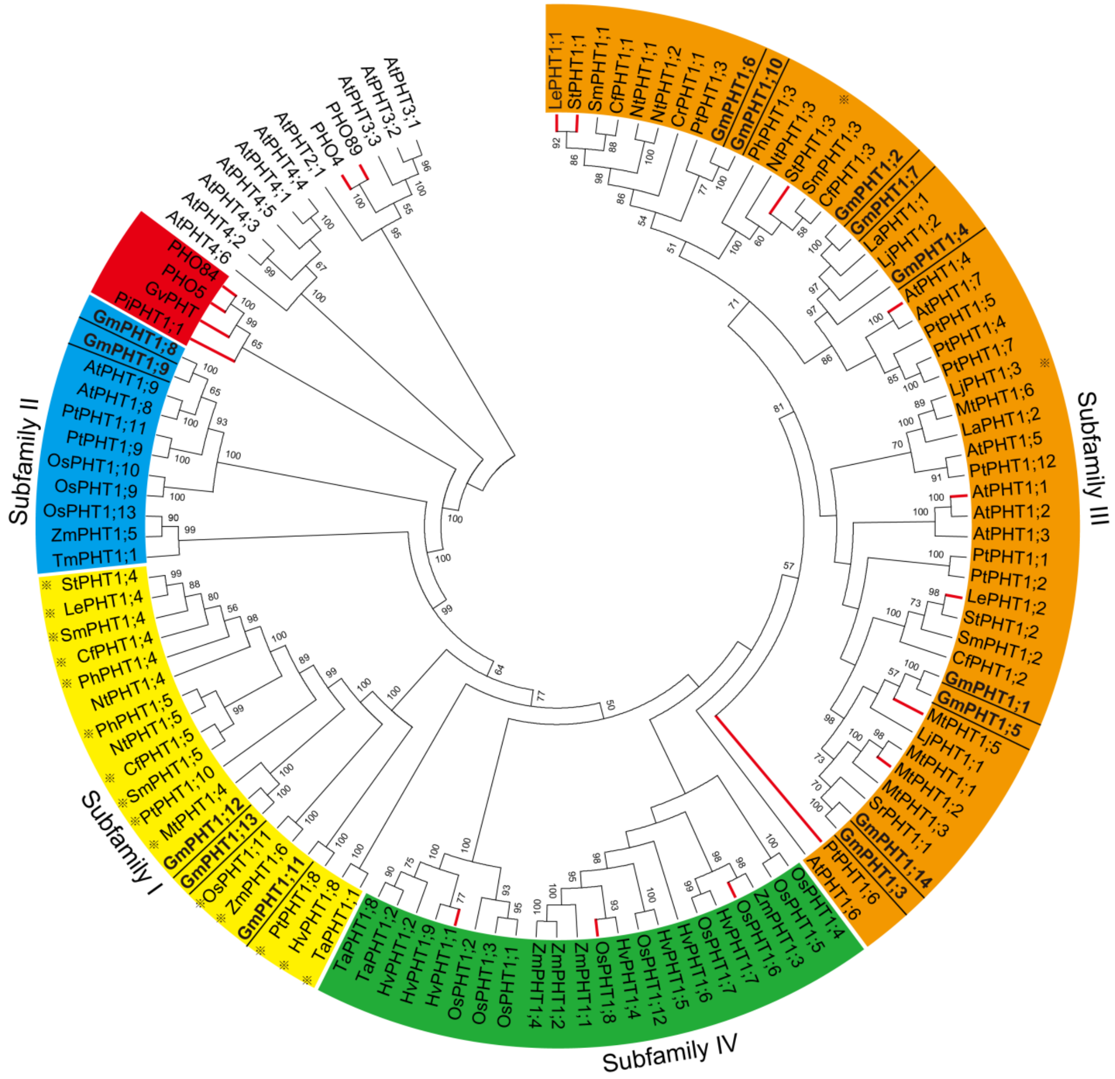

Additional file 4. PHT1 phylogenetic tree. The out-groups were AtPHT2, AtPHT3, AtPHT4 and yeast PHO89 and *N. crassa* PHO4 (clear background). The PHT1 family in fungi is shown with a red background, the dicotyledonous and monocotyledonous subfamily II with a blue background, the AM-induced di- and mono-cotyledonous subfamily I with a yellow background, the monocotyledonous subfamily IV with a green background, and the dicotyledonous subfamily III with an orange background.
