# Supplementary material for: The pattern of Phosphate transporter 1 genes evolutionary divergence in Glycine max L
Source: BMC Plant Biol. 2013 Mar 20;13:48. doi: 10.1186/1471-2229-13-48 (PMC3621523; doi:10.1186/1471-2229-13-48)
Supplement: Additional file 6 — The growth of the yeast double mutant PAM2 in the presence of various concentrations of Pi. [file 1471-2229-13-48-S6.pdf]

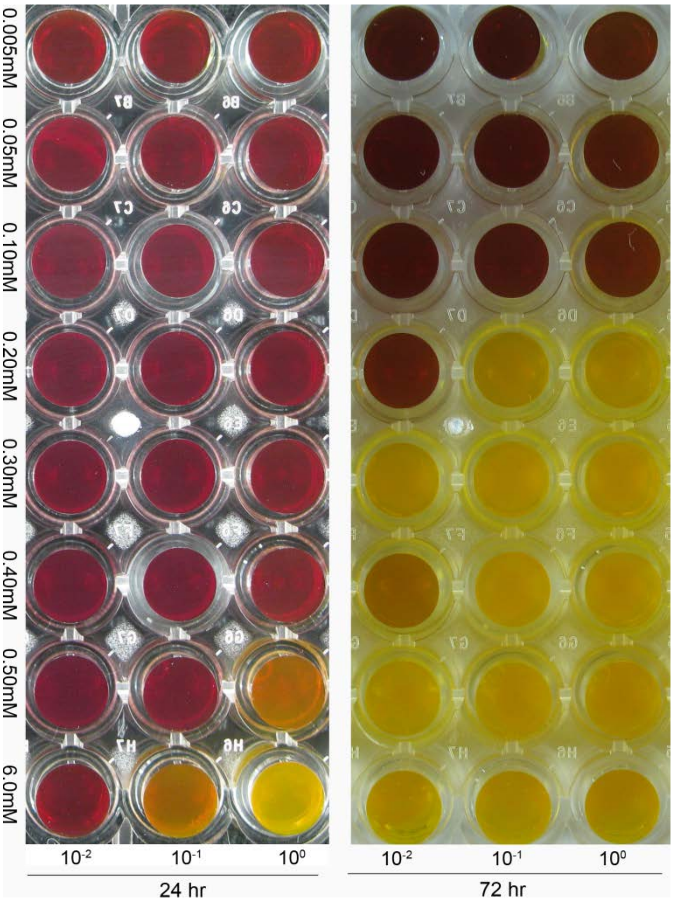

Additional file 6. The growth of the yeast double mutant PAM2 in the presence of various concentrations of Pi. The yeast cells of dilution were plated onto the solid induced medium (1 liter: 5.9 g YNB (CYN0804, ForMedium), 0.77 g mixture of amino acid without Ura, 2% galactose, 2% agar (#05038, sigma), pH6.5). Pi concentration of the induced medium was 5 μM, 50 μM, 100 μM, 200 μM, 300 μM, 400 μM, 500 μM and 6000 μM, respectively, and K^+ was supplemented with the equivalent KCl. Bromocresol purple was used as the pH indicator. Each spot represents a 5 μl aliquot of cells sampled from the indicated dilution from a master culture. Yellow color indicates the cell in the spots grew well. Images were captured after three days.
