# Supplementary material for: The pattern of Phosphate transporter 1 genes evolutionary divergence in Glycine max L
Source: BMC Plant Biol. 2013 Mar 20;13:48. doi: 10.1186/1471-2229-13-48 (PMC3621523; doi:10.1186/1471-2229-13-48)
Supplement: Additional file 7 — Transient transcription of the GmPHT1;1-YFP, GmPHT1;5-YFP and GmPHT1;6-YFP fusions in plasmolyzed onion epidermal cells. [file 1471-2229-13-48-S7.pdf]

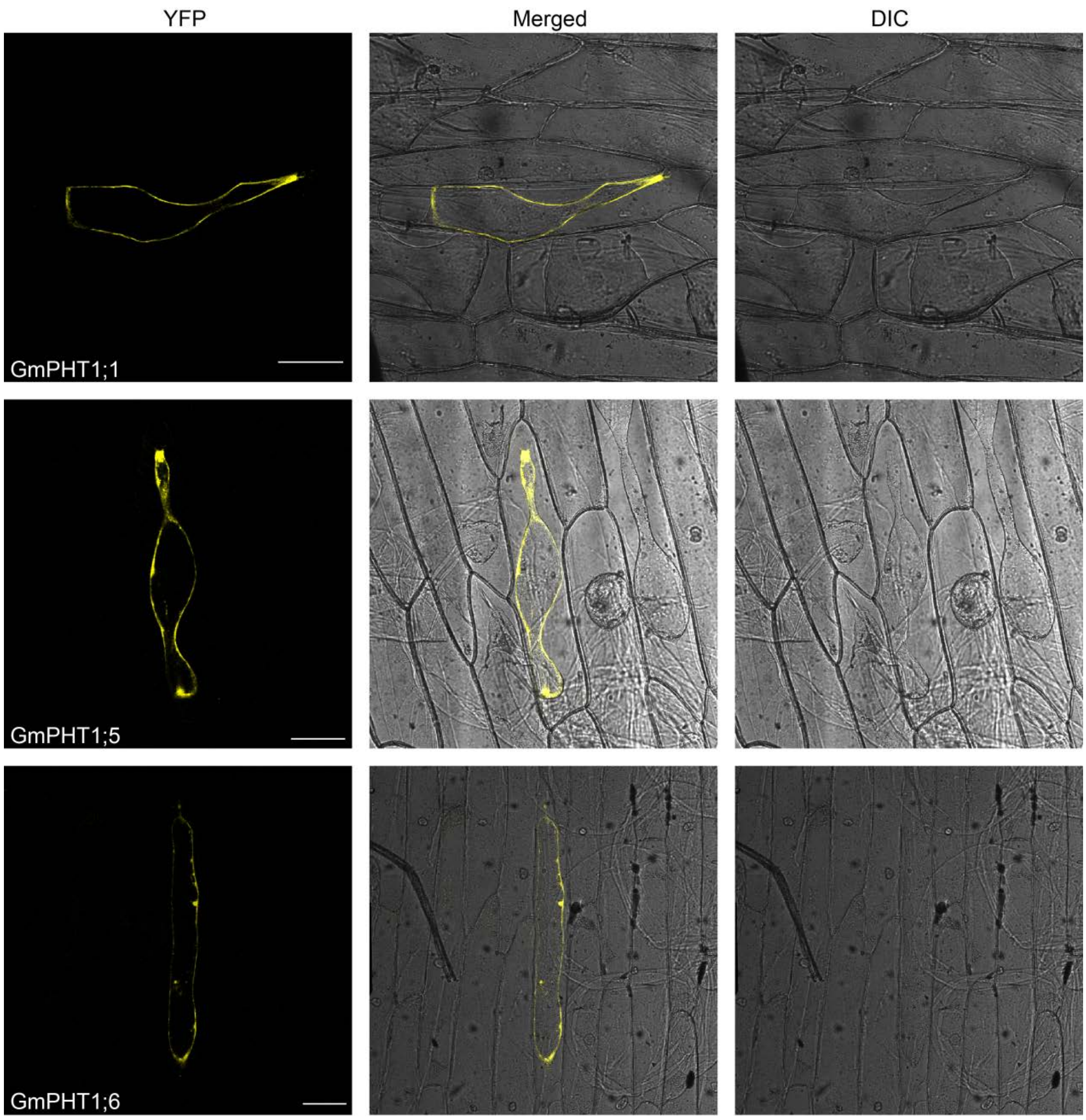

Additional file 7. Transient transcription of the *GmPHT1;1-YFP*, *GmPHT1;5-YFP* and *GmPHT1;6-YFP* fusions in plasmolyzed onion epidermal cells. DIC: differential interference contrast. Scale bar: 50 µm.
