# Supplementary material for: The pattern of Phosphate transporter 1 genes evolutionary divergence in Glycine max L
Source: BMC Plant Biol. 2013 Mar 20;13:48. doi: 10.1186/1471-2229-13-48 (PMC3621523; doi:10.1186/1471-2229-13-48)
Supplement: Additional file 8 — Motifs and domains shared by the GmPHT1 and other PHT1 proteins. [file 1471-2229-13-48-S8.pdf]

A

PHO84	: 176	GGDYPLSSIITSE
AtPHT1;1	: 142	GGDYPLSATIMSE
AtPHT1;4	: 142	GGDYPLSATIMSE
MtPT4	: 139	GGDYPLSATIMSE
GmPHT1;2	: 142	GGDYPLSATIMSE
GmPHT1;7	: 142	GGDYPLSATIMSE
GmPHT1;4	: 142	GGDYPLSATIMSE
GmPHT1;6	: 141	GGDYPLSATIMSE
GmPHT1;10	: 141	GGDYPLSATIMSE
GmPHT1;1	: 142	GGDYPLSATIMSE
GmPHT1;5	: 142	GGDYPLSATIMSE
GmPHT1;3	: 141	GGDYPLSATIMSE
GmPHT1;14	: 141	GGDYPLSATIMSE
GmPHT1;8	: 142	GGDYPLSSTIMSE
GmPHT1;9	: 142	GGDYPLSSTIMSE
GmPHT1;12	: 140	GGDYPLSATIMSE
GmPHT1;13	: 140	GGDYPLSATIMSE
GmPHT1;11	: 141	GGDYPLSAVIMSE

B

Site	TM1	TM2	TM3	TM4	TM5	TM6	TM7	TM8	TM9	TM10	TM11	TM12
Begin	80	109	136	169	206	248	346	393	421	446	486	523
End	100	129	156	189	226	268	366	413	441	466	506	543
Begin	17	72	100	123	170	213	294	346	374	403	443	483
End	37	92	120	143	190	233	314	366	394	423	463	503
Begin	17	72	100	124	170	213	295	347	373	404	444	484
End	37	92	120	144	190	233	315	367	393	424	464	504
Begin	14	69	98	131	167	210	292	342	369	398	435	469
End	34	89	118	151	187	230	312	362	389	418	455	489
Begin	17	72	100	123	170	213	291	343	369	400	442	480
End	37	92	120	143	190	233	311	363	389	420	462	500
Begin	17	72	100	123	170	213	292	344	370	401	443	481
End	37	92	120	143	190	233	312	364	390	421	463	501
Begin	17	72	100	123	170	213	291	343	369	400	442	480
End	37	92	120	143	190	233	311	363	389	420	462	500
Begin	17	71	99	123	161	212	293	346	372	400	442	470
End	37	91	119	143	181	232	313	366	392	420	462	490
Begin	17	71	99	123	161	212	293	346	372	400	442	470
End	37	91	119	143	181	232	313	366	392	420	462	490
Begin	17	72	100	124	169	213	295	347	373	401	446	483
End	37	92	120	144	189	233	315	367	393	421	466	503
Begin	17	72	100	124	169	213	295	347	373	401	446	483
End	37	92	120	144	189	233	315	367	393	421	466	503
Begin	16	71	99	122	161	212	294	346	372	403	445	483
End	36	91	119	142	181	232	314	366	392	423	465	503
Begin	16	71	100	123	161	212	294	346	372	403	443	483
End	36	91	120	143	181	232	314	366	392	423	463	503
Begin	15	67	99	133	171	199	282	329	357	389	429	465
End	35	87	119	153	191	219	302	349	377	409	449	485
Begin	15	67	98	121	168	199	282	329	357	389	429	465
End	35	87	118	141	188	219	302	349	377	409	449	485
Begin	14	70	100	132	160	211	293	343	370	397	444	469
End	34	90	120	152	180	231	313	363	390	417	464	489
Begin	14	70	100	132	160	211	293	343	370	397	445	469
End	34	90	120	152	180	231	313	363	390	417	465	489
Begin	24	71	99	123	161	212	293	345	371	411	452	486
End	44	91	119	143	181	232	313	365	391	431	472	506

Additional file 8. Motifs and domains shared by the GmPHT1 and other PHT1 proteins. A, The alignment of PHT1 signature motifs. B, The sites of predicted transmembrane (TM) domains by TopPred 2 (http://bioweb.pasteur.fr/seqanal/interfaces/toppred.html).
