# Supplementary material for: The pattern of Phosphate transporter 1 genes evolutionary divergence in Glycine max L
Source: BMC Plant Biol. 2013 Mar 20;13:48. doi: 10.1186/1471-2229-13-48 (PMC3621523; doi:10.1186/1471-2229-13-48)
Supplement: Additional file 9 — The appearance of soybean roots in plants subjected to different Pi concentration conditions. [file 1471-2229-13-48-S9.pdf]

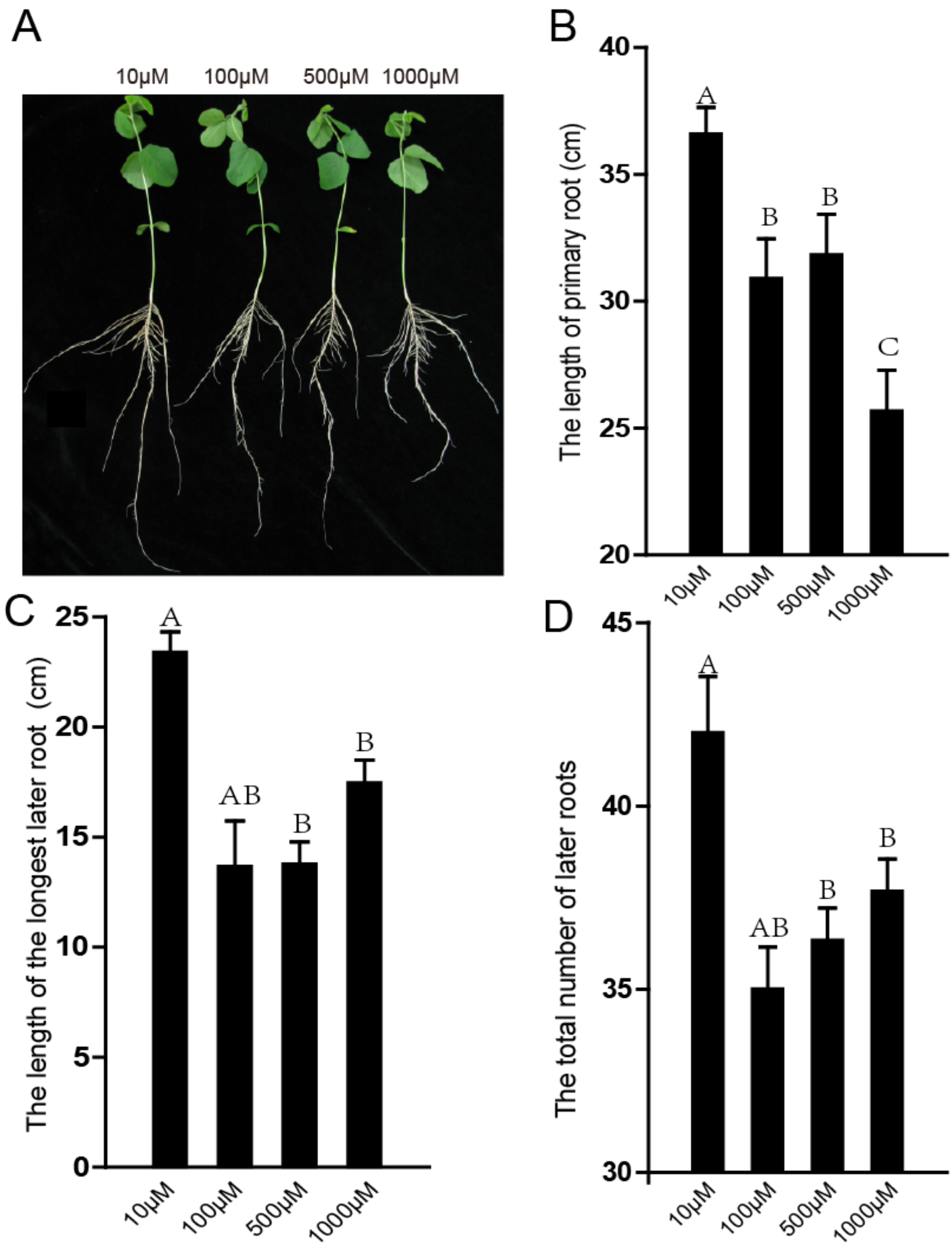

Additional file 9. The appearance of soybean roots in plants subjected to different Pi concentration conditions. A, The appearance of soybean roots. B, The statistical analysis of the length of primary root. C, The statistical analysis of the length of the longest later root. D, The statistical analysis of the total number of later roots.
